# Inhibition of APE1/Ref-1 for Neovascular Eye Diseases: From Biology to Therapy

**DOI:** 10.3390/ijms221910279

**Published:** 2021-09-24

**Authors:** Gabriella D. Hartman, Nathan A. Lambert-Cheatham, Mark R. Kelley, Timothy W. Corson

**Affiliations:** 1 Department of Ophthalmology, Eugene and Marilyn Glick Eye Institute, Indiana University School of Medicine, Indianapolis, IN 46202, USA; gdhartma@iu.edu (G.D.H.); lambertcheatham@gmail.com (N.A.L.-C.); mkelley@iu.edu (M.R.K.); 2 Stark Neurosciences Research Institute, Indiana University School of Medicine, Indianapolis, IN 46202, USA; 3 Herman B Wells Center for Pediatric Research, Department of Pediatrics, Indiana University School of Medicine, Indianapolis, IN 46202, USA; 4 Department of Pharmacology and Toxicology, Indiana University School of Medicine, Indianapolis, IN 46202, USA; 5 Department of Biochemistry and Molecular Biology, Indiana University School of Medicine, Indianapolis, IN 46202, USA; 6 Indiana University Simon Comprehensive Cancer Center, Indiana University School of Medicine, Indianapolis, IN 46202, USA

**Keywords:** redox effector factor 1, apurinic/apyrimidinic endonuclease, redox signaling, angiogenesis, inflammation, oxidative stress, retina, choroid, neovascularization, NF-κB

## Abstract

Proliferative diabetic retinopathy (PDR), neovascular age-related macular degeneration (nvAMD), retinopathy of prematurity (ROP) and other eye diseases are characterized by retinal and/or choroidal neovascularization, ultimately causing vision loss in millions of people worldwide. nvAMD and PDR are associated with aging and the number of those affected is expected to increase as the global median age and life expectancy continue to rise. With this increase in prevalence, the development of novel, orally bioavailable therapies for neovascular eye diseases that target multiple pathways is critical, since current anti-vascular endothelial growth factor (VEGF) treatments, delivered by intravitreal injection, are accompanied with tachyphylaxis, a high treatment burden and risk of complications. One potential target is apurinic/apyrimidinic endonuclease 1/reduction-oxidation factor 1 (APE1/Ref-1). The multifunctional protein APE1/Ref-1 may be targeted via inhibitors of its redox-regulating transcription factor activation activity to modulate angiogenesis, inflammation, oxidative stress response and cell cycle in neovascular eye disease; these inhibitors also have neuroprotective effects in other tissues. An APE1/Ref-1 small molecule inhibitor is already in clinical trials for cancer, PDR and diabetic macular edema. Efforts to develop further inhibitors are underway. APE1/Ref-1 is a novel candidate for therapeutically targeting neovascular eye diseases and alleviating the burden associated with anti-VEGF intravitreal injections.

## 1. Introduction

Angiogenesis, the division and proliferation of endothelial cells, causes an increase in blood vessel growth. Although angiogenesis is essential for development, abnormal angiogenesis can also occur. This is referred to as neovascularization and factors such as ischemia, hypoxia and inflammation can influence the development of neovascularization. Retinal and choroidal neovascularization almost always impairs function and can cause blindness in a wide variety of diseases, including proliferative diabetic retinopathy (PDR), neovascular age-related macular degeneration (nvAMD) and retinopathy of prematurity (ROP) [1,2,3]. Globally, it is estimated that 187 million people have AMD, while 126.6 million people have DR [4,5]. With age being a key risk factor for AMD and DR, these numbers are expected to rise as the global median age, life expectancy and population continue to increase. In addition, an estimated 185,000 premature babies developed ROP worldwide in 2010 and an estimated 32,000 neonates become blind due to ROP every year [6,7]. 

**Figure 1:**
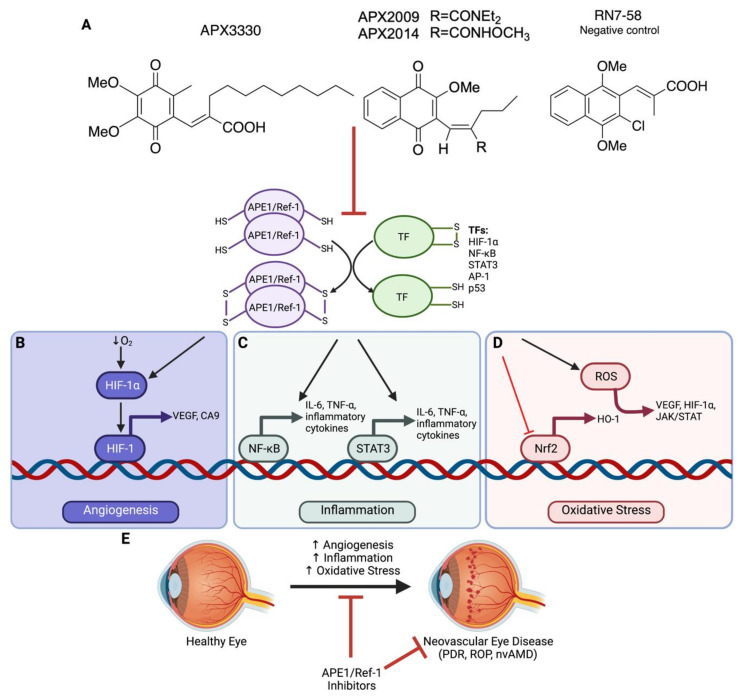
Role of APE1/Ref-1 in neovascular eye diseases. (**A**) APE1/Ref-1 redox function, inhibited by APX3330, APX2009 and APX2014, serves to regulate many transcription factors by reducing disulfide bonds of transcription factors to induce expression of various genes that play roles in angiogenesis, inflammation, oxidative stress and other pathways. APE1/Ref-1-regulated transcription factors that are linked to neovascular eye disease include HIF-1α, NF-κB, STAT3, AP-1 and p53. Analog RN7-58 is similar to APX3330, APX2009 and APX2014 in structure, but it does not inhibit redox function. (**B**) APE1/Ref-1 redox activity and low oxygen levels induce expression of HIF-1α, which can then dimerize with HIF-1β to form HIF-1. HIF-1 can then induce the expression of genes, including VEGF and CA9. (**C**) NF-κB and STAT3 are transcription factors that can be activated by APE1/Ref-1 redox activity, are involved in inflammation and can induce expression of TNF-α, IL-6 and other inflammatory cytokines. (**D**) Transcription factor Nrf2, repressed by APE1/Ref-1 in many cell types, regulates cellular defenses against oxidative stress and induces expression of HO-1 and other proteins involved in detoxification and elimination of ROS. ROS can also induce oxidative stress by inducing expression of VEGF, HIF-1α and the JAK/STAT pathway, making these various pathways connected. (**E**) Increases in angiogenesis, inflammation and oxidative stress in a healthy eye can lead to neovascular eye diseases, such as PDR, ROP and nvAMD. APE1/Ref-1 inhibitors, such as APX3330, APX2009 and APX2014, block the redox ability of APE1/Ref-1 to oxidize transcription factors. Blocking APE1/Ref-1 redox activity in the eye can decrease angiogenesis, inflammation and oxidative stress via de-repressing Nrf2. Therefore, APE1/Ref-1 redox inhibitors interfere with pathways implicated in neovascular eye diseases such as PDR, ROP and nvAMD. Created with BioRender.com. Abbreviations: TF(s), transcription factors; HIF-1α, subunit of hypoxia-inducible factor 1; HIF-1, hypoxia-inducible factor 1; STAT3, signal transducer and activator of transcription 3; NF-κB, nuclear factor κ light-chain-enhancer of activated B cells; TNF-α, tumor necrosis factor alpha; IL-6, interleukin 6; VEGF, vascular endothelial growth factor; Nrf2, nuclear factor erythroid 2-related factor 2; HO-1, heme-oxygenase 1; CA9, carbonic anhydrase 9; JAK/STAT, Janus kinase/signal transducer and activator of transcription; PDR, proliferative diabetic retinopathy; nvAMD, neovascular age-related macular degeneration; ROP, retinopathy of prematurity.

Current pharmacological treatments for retinal and choroidal neovascularization include biologic therapies targeting the vascular endothelial growth factor (VEGF) signaling pathway, but many patients experience worsened eye disease regardless of the anti-VEGF treatment. This corroborates research indicating that multiple pathways are involved in ocular angiogenesis and targeting other pro-angiogenic pathways may be the future of retinal and choroidal neovascularization medicine [8]. One potential target is apurinic/apyrimidinic endonuclease 1/reduction-oxidation factor 1 (APE1/Ref-1), a multifunctional protein that has both an endonuclease role and a redox-transcription activator role. Targeting APE1/Ref-1 with redox inhibitors may be a promising therapy for neovascular eye disease, as redox activity influences angiogenesis, inflammation, stress responses and cell survival [9]. This review explores the redox-regulated transcription factor activity of APE1/Ref-1 and how its effects on inflammation, angiogenesis and other cellular targets make it a potential candidate for therapeutically modulating neovascular eye diseases, such as nvAMD, PDR and ROP, and the vascular leakage of diabetic macular edema (DME). A small molecule APE1/Ref-1 inhibitor is already in clinical trials. Efforts to develop further inhibitors are underway, as agents such as APE1/Ref-1 redox inhibitors may modulate multiple pathways to have a greater therapeutic effect than current treatments for retinal and choroidal neovascularization (Figure 1).

## 2. APE1/Ref-1

APE1/Ref-1 (also known as Ref-1, APE, APEX, HAP1, or hAPE) was first described in early work in which an endodeoxyribonuclease that acted on apurinic/apyrimidinic (AP) sites was purified from HeLa cervical cancer cells and human placenta [10,11,12,13]. Later, Curran and colleagues identified a protein that converted transcription factors (TFs) from an oxidized to reduced state functioning in transcriptional regulation. This protein, which was named redox effector factor 1 (Ref-1), turned out to be identical to human APE1 [10,14]. Thus, the endonuclease and redox-regulation roles of APE1/Ref-1 were characterized.

### 2.1. APE/Ref-1 Functions

While the two major functions of APE1/Ref-1 impact DNA base-excision repair (BER) and redox transcriptional regulation, additional activities have been ascribed, including RNA processing, DNA maintenance, RNA quality control, miRNA metabolism, regulation of stress-response gene expression and mitochondrial DNA (mtDNA) repair [10]. APE1/Ref-1′s endonuclease function regulates both short and long patch BER and accounts for 95% of all BER abasic site repairs [9]. In BER, a glycosylase removes the damaged base to create an abasic site. APE1/Ref-1 then hydrolyzes the phosphodiester backbone, allowing for DNA polymerase to incorporate the correct nucleotides [15]. Although the DNA repair function of APE1/Ref-1 has been preserved from *E. coli* to humans, the redox function is specific to mammals [16]. The redox function directs transcription factor activity via a thiol/sulfide exchange. APE1/Ref-1 reduces transcription factors to activate their DNA binding, while APE1/Ref-1 becomes oxidized and forms disulfide bonds between cysteine residues. This thiol/sulfide exchange affects gene expression and protein synthesis driven by several TFs, including NF-κB, HIF-1α, STAT3, Nrf2 and others [9,17] (Figure 1A). Additional functions of APE1/Ref-1 include cleavage of siRNA, degradation of RNA, cleavage of RNA-containing abasic sites and transcriptional repression by indirectly binding to Ca^2+^ response elements [15,18].

### 2.2. Structure of APE1/Ref-1

**Figure 2:**
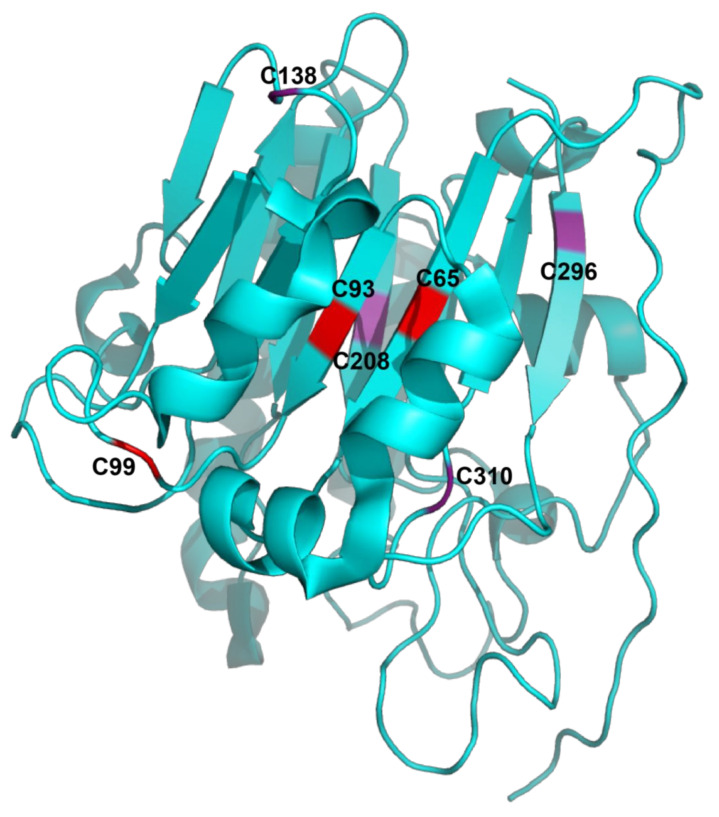
A ribbon rendering of a monomer of APE1/Ref-1 with key cysteine residues necessary for redox activity shown in red (C65, C93 and C99) and additional cysteine residues not necessary for redox activity shown in purple (C138, C208, C296 and C310). Two beta strands contain C65 and C93 and the connecting loop region contains C99. Structure: 4QHD, rendered with Mol* [24,25].

APE1/Ref-1 is a globular, α/β protein with two domains. Each domain has a 6-stranded β sheet surrounded by α helices which pack together to form a four-layered α/β sandwich [19] (Figure 2). The N-terminal region contains the nuclear localization sequence and is devoted to redox activity, while the C-terminal region exerts DNA endonuclease activity [20,21,22,23].

When proteins undergo oxidative damage, disulfide bonds form, leading to the loss of function. Redox factors such as APE1/Ref-1 can repair this oxidative damage by undergoing a thiol/sulfide exchange, causing APE1/Ref-1 to be reduced and activate specific transcription factors. Out of the seven cysteine residues in APE1/Ref-1, this thiol-mediated redox signaling depends on the formation of disulfide bonds with C65, C93 and C99 (Figure 2). Substitution of C65, C93 and C99 for alanine resulted in the inhibition of redox activity, while substitution of C138, C208, C296, or C310 resulted in the retainment of redox activity [26]. Mutations in residues C65 and C93 could result in a disruption of the hydrophobic pocket and loss of redox activity. It has also been suggested that C65 and C93 may form a disulfide bridge while in the inactive state [21]. However, compared to other redox regulatory factors such as thioredoxin, APE1/Ref-1 lacks the C-X-X-C motif required to form disulfide bonds [9,10]. It is now clear that the disulfide bridge forms between two APE1/Ref-1 proteins, as no cysteines are close enough to form intramolecular bonds [26]. Out of the cysteine residues of APE1/Ref-1, C99 and C138 are the only residues that are solvent-accessible. C65 and C93 are embedded within the protein and are not readily available for oxidation, so these residues can only act as reductants if local unfolding of APE1/Ref-1 occurs [26].

### 2.3. APE1/Ref-1 Expression

APE1/Ref-1 is ubiquitous but has differential expression among various tissues. For this review, we only discuss ocular expression of APE1/Ref-1. Under basal conditions, APE1/Ref-1 is predominantly localized to the nucleus to carry out its DNA repair function, but can also be localized to the cytoplasm and mitochondria [21,27]. APE1/Ref-1 was found to be highly expressed during retinal development, specifically in the ganglion cell layer and retinal pigment epithelium (RPE). RPE are specialized pigmented epithelium cells that lie between the retina and Bruch’s membrane and serve to maintain homeostasis of the retina and choroid and are subject to oxidative injury since they are constantly exposed to light [28]. APE1/Ref-1 accompanied the most differentiated cells in a newborn rat retina and expression increased with retinal development. These results suggested that APE1/Ref-1 is associated with retinal cell differentiation in postnatal rats [29]. APE1/Ref-1 is also highly expressed in retinal pericytes, choroidal endothelial cells and retinal endothelial cells and is upregulated in tissues where there is inflammation [28,29,30]. Furthermore, evidence suggests that secretion of APE1/Ref-1 into the extracellular milieu can act as a serological biomarker for vascular inflammation and other chronic diseases [27,31,32,33]. Acetylated APE1/Ref-1 has also been shown to be secreted from cells via the ATP binding cassette transporter A1 [33]. Given the ubiquity of APE1/Ref-1 and the associations of these various tissues with disease, it is not surprising that APE1/Ref-1 has therapeutic relevance.

## 3. Targeting APE1/Ref-1 as a Therapeutic Approach

Pharmacological interventions can be used to target either the endonuclease or redox activity of APE1/Ref-1 and several drugs targeting specific functions of APE1/Ref-1 are currently in clinical trials. The APE1/Ref-1 endonuclease inhibitor methoxyamine completed a phase I clinical trial in patients with advanced solid tumors, used in combination with temozolomide (Clinical Trials Identifier, NCT00892385) [34]. Spiclomazine, fiduxosin and SB206553 are other APE1/Ref-1 inhibitors that work by blocking the interaction of APE1/Ref-1 with NPM1, a protein involved in genomic stability, endoribonuclease activity and RNA maturation [35]. The inhibition of the APE1/Ref-1:NPM1 interaction leads to growth impairment in tumor cell lines, suggesting that APE1/Ref-1 may play an essential role in RNA quality control [35]. However, the inhibition of APE1/Ref-1 redox activity inhibits angiogenesis, inflammation, proliferation and other factors that are key contributors to neovascular eye disease. Therefore, pharmacologically targeting APE1/Ref-1 with redox inhibitors may be an avenue for the development of new therapeutics for these diseases. 

### 3.1. APE1/Ref-1 Redox Inhibitors

APX3330 ((2*E*)-2-[(4,5-dimethoxy-2-methyl-3,6-dioxo-1,4-cyclohexadien-1-yl)methylene]-undecanoic acid) (also known as E3330) is a dimethoxy benzoquinone that acts as a redox inhibitor of APE1/Ref-1 by causing increased unfolding of the APE1/Ref-1 protein and oxidation of the active disulfide residues, prohibiting the reduction of the interacting TF and keeping it in an oxidized and inactive state [36]. APX3330 (cLogP = 4.5) does not inhibit endonuclease activity of APE1/Ref-1 and blocks APE1/Ref-1 redox activation of NF-κB, HIF-1α, AP-1 and STAT3 and other TFs in vitro [17,18,37,38,39,40,41,42,43]. Previously, liquid chromatography with tandem mass spectrometry (LC-MS/MS) revealed that APX3330 increased disulfide bond formation of critical cysteine residues in APE1/Ref-1, suggesting a mechanism for its inhibitory effects on APE1/Ref-1 redox activity [41].

APX2009 ((*E*)-*N,N*-diethyl-2-((3-methoxy-1,4-dihydronaphthalen-2-yl)methylene)pentanamide) is a second-generation redox inhibitor of APE1/Ref-1. APX2009 lacks the carboxylate group and long alkyl chain that are present in APX3330, reducing its lipophilicity (cLogP = 2.7) and making it a better candidate for an orally bioavailable drug [44,45]. APX2014 (cLogP = 1.9) ((*E*)-*N*-methoxy-2-((3-methoxy-1,4-dioxo-1,4-dihydronaphthalen-2-yl)methylene)pentanamide), another second-generation redox inhibitor, also lacks the carboxylate group and long alkyl chain of APX3330, increasing its efficacy and oral bioavailability. APX2014 also more potently blocks APE1/Ref-1-induced TF DNA binding than APX3330. Both APX2009 and APX2014 were generated by structure-activity relationship studies initiating from APX3330. In addition, APX2009 showed neuronal protection in dorsal root ganglia cultures [45]. 

Other reported APE1/Ref-1 redox inhibitors include second-generation APX compounds APX2007 and APX2032. Similar to APX2009 and APX2014, these inhibitors are naphthoquinones and have a shorter lipophilic side chain. The dimethoxy moiety of APX3330 is replaced with a second aromatic ring and the carboxylic acid is replaced with carboxamide to remove the negative charge of APX3330 [15]. These modifications allow for a decrease in lipophilicity, which increases the efficacy of the second-generation compounds in comparison to first-generation APX3330. Additionally, RN8-51, RN7-60, RN10-52 and RN8-5 are other analogues of APX3330 that block the redox activity of APE1/Ref-1 [46]. Analogues RN8-51, RN7-60 and RN10-52 exhibit cell-growth inhibitor effects on ovarian SKOV-3X and HEY-C2 cancer cell lines and decrease Matrigel tube formation of erythroid-colony-forming unit cells (ECFCs) [46]. Reported natural APE1/Ref-1 redox inhibitors include resveratrol, curcumin, isoflavones genistein and daidzein and tanshinone IIA [47]. However, curcumin and isoflavones have many off-target effects [48] and resveratrol has variable bioavailability. Moreover, resveratrol showed no effect on APE1/Ref-1 redox activity in an electrophoretic mobility shift assay (EMSA) [15,46]. 

### 3.2. Specificity of APE1/Ref-1 Redox Inhibitors

While none of the natural compounds listed above have specificity for APE1/Ref-1 and have not advanced into clinical trials with APE1/Ref-1 as a target, the specificity and selectivity of APX3330 has been previously characterized. Benzoquinone and naphthoquinone analogs of APX3330 were synthesized for structure activity relationship studies and analyzed for specificity in an attempt to develop a more potent inhibitor of APE1/Ref-1. The ability of compounds to inhibit the redox function of APE1/Ref-1 was assessed in a redox EMSA assay. The benzoquinone analogs showed little improvement of activity due to significant physicochemical changes, while naphthoquinone analogs exhibited low micromolar inhibition [49]. Anthraquinones, indolequinones and dimethylhydroquinones were inactive. The importance of the quinone moiety in APX3330 was established when the naphthoquinone core was replaced by a dimethoxynaphthalene core to create RN7-58, a compound in the same series as APX3330. RN7-58 showed no effect on APE1/Ref-1 redox activity in a redox EMSA assay, despite its similarity in structure [46]. In addition, RN7-58 has been published numerous times as a negative control for biological assays [42,49,50,51]. Crucially, to demonstrate specificity of APX3330, a redox EMSA assay was used to test the ability of APX3330 to inhibit the reduction of transcription factors by thioredoxin, a cellular redox protein. Increasing amounts of APX3330 did not affect thioredoxin, supporting the specificity of APX3330 [46].

APX3330 is also effective in inhibiting the APE1/Ref-1 redox function and blocking the reduction of numerous TFs to an active state. APX3330 blocked the activation of several known TFs and their downstream targets of APE1/Ref-1 in vivo and in vitro, including STAT3, VEGF, NF-κB, MCP-1, HIF-1α, IL-6 and many others, demonstrating the target engagement of the compound via pharmacodynamic regulation [28,40,42,44,52,53,54,55,56,57,58,59,60,61]. Pharmacodynamic studies from biopsies of patients taking APX3330 exhibited downregulation of TFs regulated by APE1/Ref-1, further demonstrating target engagement and APE1/Ref-1-mediated effects in humans [62]. APX3330 also selectively binds to both recombinant APE1/Ref-1 and APE1/Ref-1 from cell nuclear extracts and displayed additional specific on-target properties, including engagement with NF-κB [9,18,26,46,63]. Taken together, the evidence demonstrates that APE1/Ref-1 redox inhibitors such as APX3330 are specific and selective inhibitors of the redox activity of APE1/Ref-1 [46].

### 3.3. APE1/Ref-1 in Cancer

APE1/Ref-1′s role in DNA repair, RNA quality control, miRNA metabolism and redox activation of transcription factors NF-κB, HIF-1α, STAT3, AP-1 and p53 has suggested that it may be a therapeutic target for many cancers [15,17,64]. Increased expression of APE1/Ref-1 is associated with cancer progression, angiogenesis and resistance to therapy in many cancers, including cancers of the pancreas, bladder, colon, prostate, lung, breast, liver, ovary, bone, malignant peripheral nerve-sheath tumors (MPNST), sarcomas and many more [15,17,26,36,65,66,67,68,69,70,71,72,73,74,75,76,77,78]. Pharmacologically inhibiting the redox function of APE1/Ref-1 may be beneficial for the treatment of various cancers, as the inhibition of APE1/Ref-1 has been associated with enhanced colorectal cancer tumor regression in preclinical studies and enhanced promyelocytic leukemia response to treatment [15,38,64]. APX3330 treatment on pancreatic cancer-associated endothelial cells (PCECs) demonstrated inhibition of tumor angiogenesis, further supporting the notion that APE1/Ref-1 redox activity may be a therapeutic target for many cancers [79]. Conversely, acetylated APE1/Ref-1 in response to hyperacetylation in triple-negative breast cancer may exert chemotherapeutic effects by amplifying intracellular programmed cell death [80].

Moreover, APX3330 is the only APE1/Ref-1 redox inhibitor to enter and complete phase I clinical trials, in which 120 mg tablets of APX3330 were given orally twice a day to patients with progressive advanced solid tumors in a dose escalation up to 600 mg/day. A total of 19 subjects received treatment and 6 subjects reported disease stabilization for over four cycles [81]. Currently, APX3330 is recommended for phase II trials for patients with progressive advanced solid tumors (Clinical Trials Identifier, NCT03375086). Taken together, this evidence suggests that the redox activity of APE1/Ref-1 may be necessary for cancer progression.

### 3.4. APE1/Ref-1 in Neuronal Diseases

APE1/Ref-1 repair activity is implicated in many neurodegenerative diseases, including Alzheimer’s disease (AD), Parkinson’s disease (PD), amyotrophic lateral sclerosis (ALS) and cerebral ischemia [17,65]. Upregulation of APE1/Ref-1 in response to oxidative stress, a key pathology of neurodegenerative disease, indicates that APE1/Ref-1 may play a role in the cellular adaptive response. Specifically, APE1/Ref-1 protein levels were elevated in nuclear extracts of the cerebral cortex of AD patients, in hippocampal cells from AD patients, in the CNS of ALS patients and in cells treated with MPP+, a toxin that can induce PD [82,83,84,85,86]. Furthermore, following cerebral ischemia, upregulation of APE1/Ref-1 protects hippocampal neuronal structure, synaptic function and viability [87]. Other evidence suggests that overexpression of APE1/Ref-1 allows for neuroprotection in recovery after ischemic injury, as APE1/Ref-1 DNA BER is essential to repair AP sites following ischemic injury [88].

APE1/Ref-1 redox activity may also be important in sensory neuropathy, as APE1/Ref-1 redox inhibitors demonstrated neuroprotective effects. Chemotherapy-induced peripheral neuropathy (CIPN), a side effect of several chemotherapeutics, results in alterations to peripheral sensory function. Use of APE1/Ref-1 redox inhibitor APX3330 resulted in an increase in DNA repair activity of APE1/Ref-1 in rats, which reduced neurotoxic effects induced by CIPN [89,90]. Further work investigated the effects of APX2009 in response to CIPN and demonstrated that APX2009 produced a neuroprotective effect against cisplatin and oxaliplatin-induced toxicity without affecting the anti-cancer activity of the platins [45]. Given APE1/Ref-1′s role in oxidative stress response in PD, AD, ALS, cerebral ischemia and sensory neuropathy, it is evident that APE1/Ref-1 modulates a wide variety of disease states via various pathways.

### 3.5. APE1/Ref-1 in Other Diseases

Factors such as AP-1, HIF-1α, NF-κB and STAT3 have been implicated in irritable bowel disease (IBD). Because APE1/Ref-1 regulates these factors, APE1/Ref-1 has also been implicated in IBD and APX3330 reduced enteric neuropathy and intestinal dysfunction [91]. APE1/Ref-1 is also increased in conditions such as ischemia, hypoxia and inflammation, and APE1/Ref-1 serum levels are elevated in mice with acute viral myocarditis, suggesting that APE1/Ref-1 plays a role in inflammatory conditions such as myocarditis [32]. Additional studies demonstrated increased APE1/Ref-1 expression in aortic endothelial cell macrophages of atherosclerotic plaques [31]. Taking this together, APE1/Ref-1 could potentially be used as a biomarker to assess the degree of vascular inflammation. APE1/Ref-1 has also been implicated elsewhere in cardiovascular disease and in gastric cellular response to *Helicobacter pylori* infection [17,65].

## 4. Neovascular Eye Diseases

Given the therapeutic relevance of APE1/Ref-1 in a plethora of disease states, APE1/Ref-1 has also been implicated in several neovascular eye diseases. Posterior ocular neovascularization can arise from either the retinal or choroidal vasculature. Retinal neovascularization occurs in PDR and ROP, while choroidal neovascularization occurs in nvAMD. In both cases, neovessels, which lack tight junctions, leak plasma into surrounding tissue, disrupting retinal and choroidal architecture [92]. This disruption ultimately leads to a significant decline in vision. APE1/Ref-1 targets many pathways associated with neovascularization, making it a potential therapeutic target for these neovascular posterior eye diseases.

### 4.1. Age-Related Macular Degeneration

AMD is the leading cause of irreversible vision loss in those over the age of 65 and costs USD 4.6 billion annually in direct healthcare costs in the United States alone [93,94,95]. Currently, it is estimated that 11 million people in the United States and 187 million people globally have AMD [4,95]. By 2040, the population of those affected with AMD is expected to increase to 288 million [4,96].

There are two types of AMD—dry AMD and nvAMD. Dry AMD accounts for 90% of all AMD, while nvAMD accounts for about 10% of all AMD cases but the majority of AMD-related blindness. Dry AMD is characterized by the accumulation of drusen above Bruch’s membrane, leading to the degeneration of the RPE and photoreceptors and causing vision loss [95,96,97]. nvAMD is characterized by neovascularization in which neovessels from the choroid invade the outer retina, subretinal space, or sub-RPE space, causing a decrease in central vision [98]. Plasma leakage from these neovessels results in ischemia which leads to the production of VEGF to further stimulate neovascularization [97]. Pathways including inflammation, lipid metabolism, cellular toxicity, oxidative stress and extracellular matrix (ECM) maintenance are all implicated in AMD pathogenesis [96].

Risk factors for AMD include aging, genetic susceptibility and environmental risk factors, such as smoking, physical activity, diet, high body mass index (BMI) and diabetes [96,99]. There are, as of yet, no approved therapies for dry AMD. However, because VEGF is a key stimulator of neovascularization, intravitreal (IVT) injections of pharmaceutical agents that target the VEGF pathway are the first-line treatment for nvAMD (see Section 4.4). Other treatment options include laser photocoagulation and photodynamic therapy. Pegaptanib was the first VEGF-A inhibitor approved by the FDA for treatment of AMD in 2004 and works by binding and inhibiting the isoform VEGF_165_. Other pharmacological agents that target the VEGF pathway and are currently used for AMD treatment in the United States include ranibizumab, bevacizumab, aflibercept and brolucizumab [100]. 

### 4.2. Diabetic Retinopathy and Diabetic Macular Edema

DR is the leading cause of blindness among working age adults and affects an estimated 126.6 million people worldwide [5,101]. Pathogenesis of diabetic retinopathy begins when chronic hyperglycemia due to diabetes produces advanced glycation end products, which can lead to retinal capillary occlusion, microaneurysms, macular edema and ischemia [92,102,103]. This initial stage is often referred to as non-proliferative DR (NPDR) [104]. Then, in response to hypoxia, the RPE, pericytes, endothelial cells, retinal ganglion cells and Müller glia produce VEGF, leading to the induction of pro-inflammatory cytokines, such as TNF-α, IL-6 and IL-1β, which induce neovascularization. This neovascular stage is referred to as proliferative DR (PDR) [104,105,106]. One of the most common causes of vision loss in both NPDR and PDR is the leakage of fluid into the macula, termed diabetic macular edema (DME) [104]. 

Risk factors for DR include hyperglycemia, hypertension, dyslipidemia, high BMI, low levels of physical activity and insulin resistance [102]. Current treatments include the use of anti-VEGF agents, which improve visual acuity in 25–30% of DR patients [102,107,108]. Anti-VEGF therapy for DME results in a decrease in fluid leakage and can be sufficient to restore vision in DME patients [106,109]. Laser photocoagulation is also used for treatment of PDR and DME [104]. 

### 4.3. Retinopathy of Prematurity

ROP is a vaso-proliferative retinopathy affecting premature infants that can result in blindness or visual impairment. The incidence of ROP in the United States increased from 14.7% in 2000 to 19.9% in 2012 [110]. Globally, ROP is the biggest threat for vision in premature infants and 68% of infants born weighing less than 1251 g develop ROP [7,111]. Many premature infants are placed in high oxygen incubators to assist with respiration, since premature infants’ lungs are not fully developed and need supplemental oxygen [112]. However, this hyperoxic environment causes downregulation of retinal VEGF and vaso-obliteration of the developing vasculature of the retina. When the infants are returned to normal oxygen conditions, low relative oxygen levels cause an increase in VEGF expression, which drives neovascularization of the retina [7,103,112,113].

Prematurity and low birth weight are the greatest risk factors for ROP [7,112]. Assisted ventilation for longer than one week, high blood-transfusion volumes, low caloric intake, hyperglycemia, insulin therapy and anemia are other risk factors [112]. Current treatments for ROP include laser photocoagulation of the retina to slow the abnormal blood vessel growth. However, laser photocoagulation for ROP has been associated with corneal edema, intraocular hemorrhage and cataract formation. Because VEGF is a key cytokine implicated in ROP, use of anti-VEGF drugs such as bevacizumab has become widely used for treatment of ROP [114]. However, concerns still remain about the safety of anti-VEGF compounds for ROP, particularly the effects bevacizumab may have on neurodevelopment and systemic toxicity. To address bevacizumab systemic toxicity concerns, a recent study indicates that low-dose bevacizumab treatment for ROP results in positive retinal structure outcomes, but many patients require additional treatment [115,116]. Future studies will need to be conducted to evaluate the effects bevacizumab may have on neurodevelopment.

### 4.4. Shortcomings of Current Therapies

Current pharmacological interventions for nvAMD, PDR/DME and ROP target the VEGF signaling pathway with IVT injections of bevacizumab, ranibizumab, pegaptanib, aflibercept and brolucizumab [100]. Other neovascular eye diseases currently treated with anti-VEGF therapy include retinal vein occlusion, myopic choroidal neovascularization, neovascular glaucoma, central serous retinopathy, ocular tumors, corneal neovascularization and more [117]. These drugs are usually delivered via an injection into the vitreous humor of the eye in intervals of 4–16 weeks and often continue for life [118]. Anti-VEGF therapy has reduced the rate of legal blindness by over 50% in those affected with nvAMD and PDR regresses as early as 24 h after the first bevacizumab injection [119,120].

Despite these positive outcomes, there are concerns surrounding the safety and efficacy of anti-VEGF therapy for neovascular eye diseases. Many patients become resistant to anti-VEGF therapy and develop tachyphylaxis, or diminished response to medication. Resistance may be due to alterations in neovascular architecture, compensatory mechanisms, alternative angiogenic pathways, genetic variations, or tolerance to anti-VEGF drugs [8,121]. In addition, anti-VEGF drugs have the potential to cause systemic side effects, such as myocardial infarction, stroke, delayed wound healing and non-ocular hemorrhage [122]. One study using health care databases in Ontario, Canada, observed a 1.74-fold increase in the rate of systemic thromboembolic emergencies in over 57,000 patients 1 year after receiving IVT injections of bevacizumab or ranibizumab [123]. Furthermore, treatment responses to anti-VEGF therapy vary between patients and not all patients experience stable vision improvements in response to therapy [96,100]. Because of this, there is a need for alternative treatment options that target other pathways in order to improve patient outcomes.

IVT injections are the standard of care delivery route for anti-VEGF biologics, but these injections are associated with high treatment burden on both the patient and physician [121]. IVT injections are labor-intensive, cause the patient discomfort and have the potential to result in rare but serious complications, such as endophthalmitis, intraocular hemorrhage, cataract, glaucoma, inflammation, or retinal detachment [96,124,125]. In a study that surveyed patient preferences in retinal drug delivery, the majority of respondents reported willingness to receive IVT injections in order to preserve vision, but would prefer alternative drug delivery methods, such as eye drops or oral tablets [126]. This highlights the significance of developing alternative drug delivery methods to increase patient comfort and accessibility to treatment.

Because of tachyphylaxis and the potential for the development of serious complications from IVT injections of anti-VEGF biologics, new therapies that bypass IVT injections and target multiple pathways may be the future of treatment for neovascular eye diseases. Agents that modulate multiple pathways may have a greater therapeutic effect on disease states such as nvAMD, PDR, DME and ROP, since the pathogeneses are so complex for these diseases. Targeting the redox function of APE1/Ref-1 may be a promising therapeutic approach, since APE1/Ref-1 redox activity affects multiple downstream pathways associated with neovascular eye disease, including angiogenesis, inflammation, oxidative stress response and cell-cycle control.

## 5. APE1/Ref-1 and Angiogenesis in Neovascular Eye Disease

Abnormal angiogenesis of the retinal and choroidal vasculature requires endothelial cell migration, proliferation and degradation of the ECM and can result in loss of vision or blindness [127]. The redox activity of APE1/Ref-1 regulates many transcriptional targets that are involved in ocular angiogenesis and inflammation, such as HIF-1α and its downstream target VEGF, STAT3, NF-κB and others [9,15,78] (Figure 1B,C).

### 5.1. Role of HIF-1

HIF-1 is a heterodimeric transcription factor that regulates cellular response to hypoxia and is implicated in many ocular diseases, such as PDR, nvAMD and ROP. When oxygen is plentiful, a prolyl hydroxylase hydroxylates highly conserved proline residues on HIF-1α, which promotes the binding of HIF-1α to the von Hippel–Lindau protein (pVHL). When this coupling takes place, an E3-ubiquitin ligase complex associates and targets HIF-1α for degradation [128]. In response to hypoxia, the prolyl hydroxylase is inactivated, allowing HIF-1α to dimerize with HIF-1β to form HIF-1 [129]. Then, HIF-1 translocates to the nucleus and recognizes hypoxia response elements, leading to the upregulation of many pro-angiogenic genes such as VEGF, angiopoietin-2, VE-PTP, CXCR4, PDGF-B, CA9 and other factors that are implicated in retinal and choroidal neovascularization [9,129,130]. The inhibition of HIF-1 results in a reduction in VEGF, CA9 and other proangiogenic genes [9,15]. Redox signaling via APE1/Ref-1 modulates HIF-1 by increasing the transcriptional activity of HIF-1 [36,40,74]. Following the induction of HIF-1, CA9 responds to hypoxia by coordinating with a bicarbonate transporter to regulate intracellular pH [17,50]. APE1/Ref-1 redox signaling also modulates HIF-1-mediated CA9 expression. Although this relationship has not been conclusively shown in the eye, previous studies revealed that reduction of APE1/Ref-1 redox signaling with APX3330 reduces CA9 expression in PDAC cells, demonstrating that APE1/Ref-1 contributes to HIF-1-mediated transcription [74].

### 5.2. Role of VEGF

VEGF, a downstream target of HIF-1 signaling, is upregulated in response to hypoxia and is important in the development and maintenance of blood vessels [129]. In the eye, RPE-derived VEGF expression increases retinal vascular permeability of the choriocapillaris [124,129,131]. When APE1/Ref-1 was overexpressed in human umbilical vein endothelial cells (HUVECs), VEGF secretion and *VEGF* mRNA expression were increased. When HUVECs were treated with APX3330, *VEGF* mRNA expression decreased, suggesting that the redox function of APE1/Ref-1 is important for the induction of VEGF [54]. 

### 5.3. Suppression of Angiogenesis In Vitro with APE1/Ref-1 Inhibitors

The redox activity of APE1/Ref-1 is essential for angiogenesis in vitro in a variety of cell types. APE1/Ref-1 inhibitors APX3330, APX2009 and APX2014 have been studied in the context of retinal and choroidal angiogenesis in vitro (Table 1). APX3330 dose-dependently decreased angiogenesis of murine retinal vascular endothelial cells (RVECs) and a macaque choroidal endothelial cell-like cell line (Rf/6a) by suppressing proliferation, migration and tube formation [30,55]. In addition, dual treatment of APX3330 with bevacizumab showed an additive decline in tube formation and proliferation in Rf/6a cells [55]. Second generation APE1/Ref-1 inhibitors APX2009 and APX2014 dose-dependently decreased choroidal sprouting, proliferation, tube formation and endothelial cell migration of human retinal microvascular endothelial cells (HRECs) and Rf/6a cells [44]. Because migration, proliferation and organization of endothelial cells are essential to angiogenesis, these results indicate that APX3330, APX2009 and APX2014 have anti-angiogenic activity.

### 5.4. Suppression of Angiogenesis In Vivo with APE1/Ref-1 Inhibitors 

The redox activity of APE1/Ref-1 is also essential for neovascularization in vivo in *Vldlr^−/−^* mice and in the laser-induced CNV model (Table 1). *Vldlr^−/−^* mice lack the very low-density lipoprotein receptor and spontaneously develop subretinal neovascularization (Type III CNV). *Vldlr^−/−^* mice have higher expression of APE1/Ref-1 in the retina than wild-type littermates and APX3330 decreased neovascularization in *Vldlr^−/−^* mice [30]. Following L-CNV (Type II CNV), a single IVT injection of APX3330 for a final intraocular concentration of 20 μM suppressed L-CNV lesion area [28]. Additionally, intraperitoneal (IP) administration of APX3330 twice a day at 50 mg/kg for 5 days on and 2 days off for two weeks reduced L-CNV volume by 25% [44]. IP injection (25 mg/kg twice daily for two weeks) of second-generation APE1/Ref-1 inhibitor APX2009 also decreased L-CNV volume without causing systemic or ocular toxicity, as APX2009 treatment did not affect normal retinal vasculature aside from lesions induced by laser nor did it affect mouse weights [44]. These results demonstrated, for the first time, the effectiveness of systemic administration of APE1/Ref-1 inhibitors at decreasing L-CNV lesion size.

**Figure 3:**
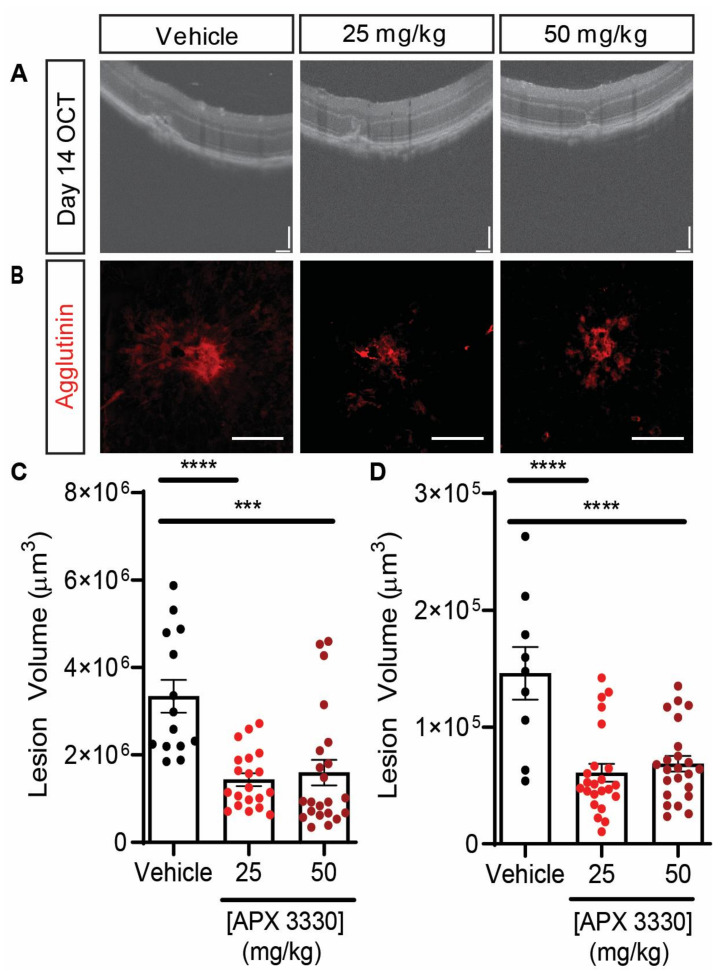
APX3330 gavage efficacy in a laser-induced CNV model. C57BL/6J mice (7–8-week-old, female) received laser-induced CNV induction using a Micron IV laser injector, then received twice daily gavage for 14 days of 25 mg/kg APX3330, 50 mg/kg APX3330, or ethanol/Cremophor vehicle. (**A**) Representative OCT images of lesions imaged 14 days post-laser. Scale bars = 100 μm. (**B**) Representative confocal images of agglutinin-stained choroidal flat mounts following enucleation 14 days post-laser. Scale bars = 100 μm. (**C**) Quantification of OCT lesion volume [134] (represented in A) using ImageJ software. (**D**) Quantification of confocal, z-stack agglutinin staining images (represented in B) using ImageJ software. Results demonstrated a significant decrease in lesion size for both 25 mg/kg and 50 mg/kg APX3330 compared to vehicle (***, *p* = 0.0002, ****, *p* = 0.0001, ANOVA with Dunnett’s post-hoc test). Mean ± SEM for individual lesions (2–4 per eye) from *n* = 5 mice per group shown.

To further test other routes of administration of APX3330, we examined the effects of APX3330 as a treatment for L-CNV when administered by gavage, as pharmacokinetic studies suggested that APX3330 may be more efficacious when administered orally. Mice received either 25 mg/kg or 50 mg/kg gavage of APX3330 twice daily for 14 days. Results demonstrated a significant decrease in lesion volume for both doses compared to vehicle (Figure 3). Moreover, no retinal degeneration was seen in eyes after oral APX3330 treatment. The lesion volume decreased by more than 50% when APX3330 was administered by gavage, compared to previous results that demonstrated a reduction in lesion size by about 25% when APX3330 was administered by IP injection. Comparatively, IVT injection of murine anti-VEGF antibody demonstrates a 30–50% decrease in CNV lesion volume [132,133]. Therefore, these results suggest that oral administration of APX3330 may be a superior route of administration to IP injections to reduce lesion size, indicating that APE1/Ref-1 redox inhibitors such as APX3330 may be potential oral therapeutics for neovascular eye diseases.

## 6. APE1/Ref-1 and Inflammation in Neovascular Eye Disease

Inflammation is a biologic response to injury and chronic inflammation can induce tissue damage. Inflammation plays a role in the development and pathogenesis of many ocular diseases, such as AMD, ROP and DR. In the vitreous of patients affected with retinopathies, increased levels of pro-inflammatory molecules, including TNF-α, IL-6, IL-8, MCP-1, endothelin 1, VEGF and ICAM-1, have been observed [135,136]. Intracellular APE1/Ref-1 is transiently increased in response to inflammation [9]. Specifically, APE1/Ref-1 regulates the transcriptional activity of NF-κB, STAT3, growth factors and cytokines by modulating DNA-binding activity. APE1/Ref-1 inhibitors abrogate the DNA-binding activity of these TFs, indicating that APE1/Ref-1 may play a role in inflammation in neovascular eye disease (Figure 1C) [37,43,52]. Since NF-κB and STAT3 are redox-sensitive, APE1/Ref-1 may regulate the transcriptional activity of these pro-inflammatory molecules by maintaining the reduction of the cysteine residues [9].

### 6.1. Role of NF-κB

NF-κB activates the transcription of genes involved in proliferation, migration and invasion and contributes to the inflammatory response. In canonical NF-κB signaling, a variety of signaling kinases activate the IκB kinase (IKK) complex. Then, the IKK complex phosphorylates IκBα on select serine residues, signaling for proteasomal degradation of IκBα. This degradation releases NF-κB homo- or heterodimers to translocate to the nucleus and promote the transcription of target genes [137,138]. The molecular components of the NF-κB dimer can include RelA (p65), p50, p52 and others [139]. Activation of pro-inflammatory nuclear transcription factors such as NF-κB results in an increased expression of cytokines, chemokines, VCAM-1, VEGF and other molecules that contribute to the pro-inflammatory milieu [105]. The inhibition of NF-κB results in the reduction of IL-6, IL-8, TNF-α and other inflammatory agents [15]. 

APE1/Ref-1 redox inhibitors suppress NF-κB expression, as APE1/Ref-1 induces NF-κB expression and contributes to the pro-inflammatory response. APX3330 inhibited DNA binding of NF-κB in a human T cell line without affecting the degradation of IκBα and phosphorylation of p65, both of which are required for NF-κB activation [52]. In the ocular context, APX3330 also suppressed NF-κB transcriptional activity in Rf/6a cells, as 80 and 100 μM doses of APX3330 effectively reduced p65 expression [55]. Second-generation APE1/Ref-1 inhibitors APX2009 and APX2014 dose-dependently reduced translocation of the p65 subunit of NF-κB into the nucleus in HRECs and treatment of HRECs with APX2009 and APX2014 decreased downstream mRNA targets of NF-κB including *VCAM1, CCL20* and *VEGFA* [44]. Additionally, APX3330 treatment of RPE cells effectively blocked the upsurge of NF-κB activity in response to pathological stress induced by oxidized low density lipoprotein (oxLDL), a widely used experimental reagent that triggers inflammation and pathological stress [28]. In Rf/6a cells, APX3330 also downregulated the production of MCP-1, an NF-κB transcriptionally regulated chemokine. APX3330 treatment reduced the upsurge of MCP-1 in RPE cells following oxLDL treatment, implicating APE1/Ref-1′s influence on cytokines in multiple ocular cell types and how it may be a potential target to control inflammation in neovascular eye disease [28,55]. APE1/Ref-1 also regulates various cytokines that are downstream of NF-κB and other pro-inflammatory pathways, as the inhibition of APE1/Ref-1 redox activity with APX3330 suppressed IL-5 and TNF-α production in macrophages [43]. In a vascular inflammation model in which HUVECs were treated with TNF-α, a factor closely associated with NF-κB, secreted acetylated-APE1/Ref-1 rapidly deacetylated and inhibited TNF-α-stimulated endothelial inflammation, suggesting that a conformational change in the extracellular domain of TNF receptor 1 is necessary to regulate TNF-α-stimulated inflammation in response to APE1/Ref-1 [140]. The acetylation of APE1/Ref-1 also affected the functionality of APE1/Ref-1, as acetylated APE1/Ref-1 demonstrated no redox activity, demonstrating that APE1/Ref-1 redox activity affects inflammation. On balance, these results demonstrate that APE1/Ref-1 redox inhibition suppresses NF-κB activity, implicating APE1/Ref-1′s role in the inflammatory neovascular response. 

### 6.2. Role of STAT3

In response to pro-inflammatory cytokines, growth factors and hormones, JAK phosphorylates STAT, which induces dimerization of STAT. Dimerized STAT can then translocate to the nucleus and regulate DNA binding and transcriptional activity [17,141]. STAT3 is a downstream transcriptional target of APE1/Ref-1 and STAT3 signaling promotes inflammation and angiogenesis [37]. The inhibition of APE1/Ref-1 results in the inhibition of STAT3 binding to DNA and a decrease in inflammatory cytokines, while APE1/Ref-1 overexpression results in increased transcriptional activity of STAT3 [17,37]. Treatment of pancreatic ductal adenocarcinoma cells (PDAC) with APX3330 blocked STAT3 DNA binding [37]. As little as 10 μM APX3330 reduced the STAT3 DNA-binding activity in Rf/6a cells, implicating APE1/Ref-1′s role in directing STAT3 DNA binding. Immunoblot analysis of the expression of pSTAT3 in these cells in response to APX3330 treatment revealed no significant alterations in pSTAT3 or total levels of STAT3 [55]. These results indicate that APE1/Ref-1 specifically directs STAT3 DNA binding without directly impacting total STAT3 levels. 

## 7. APE1/Ref-1 and Oxidative Stress in Neovascular Eye Disease

The retina’s high energy demand and metabolic rate makes it particularly prone to reactive oxygen species (ROS) accumulation, thereby contributing to oxidative stress and development of retinopathies [142,143]. ROS are implicated in the process of angiogenesis, as the elevation of ROS production and insufficient ROS scavenging result in the elevation of superoxide anion and hydrogen peroxide levels. Oxidative stress results in the overproduction of superoxide and suppression of antioxidant defense, which leads to mitochondrial dysfunction and induction of pro-inflammatory pathways, ultimately contributing to the development of retinopathies such as DR or ROP [144,145]. In addition, hyperglycemia, hyperlipidemia and inflammation, three hallmarks of diabetes, stimulate the production of ROS and further contribute to the development of DR [142,146]. Not only are ROS involved in the induction of oxidative stress, but ROS can also mediate angiogenesis and inflammation by inducing VEGF expression, HIF-1α signaling and activating the JAK/STAT pathway [143]. Clearly, oxidative stress can adversely affect vision and contribute to the development of many retinopathies. 

However, APE1/Ref-1 redox inhibitors may activate ROS-responsive transcription and reverse the effects of oxidative stress [147] (Figure 1D). APX3330 rescued RPE cells from oxidative stress, reducing intracellular accumulation of ROS and attenuating Nrf2/Nrf1, p53, CBF/NF-y, YY1 and MTF-1 transcription factors, all of which have been implicated in oxidative stress response [28]. Other evidence further supports APE1/Ref-1′s redox function in ROS production, as APX3330 treatment following cerium oxide nanoparticle treatment on HUVECs decreased tube formation, indicating the importance of the APE1/Ref-1 pathway in angiogenesis in a ROS-excessive environment [148].

### 7.1. Role of Nrf2

Nuclear factor erythroid-2-related factor 2 (Nrf2) is a protective transcription factor that is upregulated in response to oxidative stress. Following the induction of oxidative stress, the reduction of cysteine residue 151 of Keap1 allows for Nrf2 to dissociate from Keap1 and enter the nucleus to activate antioxidant response element (ARE) genes, which protect the cell from oxidative stress [142,143,149]. The activation of Nrf2 also prevents the overproduction of pro-inflammatory cytokines such as IL-6 and IL-1β [150]. Previous studies have suggested that the PI3K/Akt and ERK pathways are implicated in the modulation of Nrf2 expression [143,151]. Specifically, in the retina, Nrf2 has been implicated as an important cytoprotective mechanism in response to oxidative stress and may be a potential pharmacological target for retinal diseases [152,153]. In aged retinas, there is an increase in pro-inflammatory molecules and a decline in Nrf2 and heme oxygenase-1 (HO-1), suggesting poor protective response to oxidative stress, which may play a role in the pathogenesis of retinopathies [142,154]. In PDAC cells, the suppression of APE1/Ref-1 redox activity with APX3330 resulted in increased Nrf2 activity in a dose-dependent manner and did not increase ROS levels, suggesting that APE1/Ref-1 redox activity influences Nrf2 activity [39]. However, the induction of pathological stress in human RPE cells with oxLDL resulted in increased Nrf2 activity and, following treatment of APX3330, Nrf2 activity decreased [28]. The disparate effects of APE1/Ref-1 redox inhibitors on Nrf2 levels in RPE cells compared to cancer cells leave open the question on the mechanism by which APE1/Ref-1 inhibition ameliorates oxidative stress in ocular tissues, how these effects contribute to neovascularization and what is the relative role of Nrf2 in the process. This is particularly relevant as HO-1, which is Nrf2-regulated, is upregulated in response to oxidative stress, protecting cells from ROS. HO-1 expression is induced by APX3330 as discussed below. 

### 7.2. Role of HO-1

HO-1 is an Nrf2-regulated gene that catalyzes the degradation of heme and is upregulated in response to oxidative stress to protect cells from ROS accumulation [151]. The induction of HO-1 in response to Nrf2 signaling induces anti-inflammatory effects and can lead to the inhibition of NF-κB signaling; evidence suggests that the ERK and PI3K signaling pathways participate in regulating HO-1 expression [143,149,155]. In the retina, protection from oxidative damage can be aided via upregulation of HO-1 [143]. Although HO-1 expression related to APE1/Ref-1 has not yet been characterized in the eye, the inhibition of APE1/Ref-1 in PDACs with APX3330 induced HO-1 expression [39].

## 8. APE1/Ref-1 and Cell-Cycle Control in Neovascular Eye Disease

APE1/Ref-1 is crucial in regulating the cell cycle, specifically, by enabling cells to progress from the G1 to the S phase. In prostate cancer cells, treatment with APX2009 induces G1 cell-cycle arrest but does not cause cell death [57]. Similarly, the cell-cycle profiling of HRECs demonstrated that treatment with APX2009 and APX2014 blocked the induction of the S phase and increased the number of cells in the G1 phase [44]. APX2009 and APX2014 also inhibited cell proliferation in HRECs and Rf/6a cells, further supporting the notion that APE1/Ref-1 inhibitors block angiogenesis [44]. In another study, the cell index (CI) was evaluated in response to APX3330 treatment of RVECs. The CI integrates information on cell viability, number, morphology and adhesion. APX3330 reduced CI of RVECs of wildtype and *Vldlr^−/−^* mice. APX3330 was more potent at reducing CI values of RVECs than bevacizumab treatment, indicating that targeting the APE1/Ref-1 redox function may be more effective than VEGF targeting for inhibiting cell proliferation [30].

Senescence is also an important contributor to the development of AMD. When RPE cells become senescent, oxidative damage and stress can increase, ultimately promoting AMD [156]. Treatment of ARPE-19 cells with APX3330 protected the cells from a stress-induced senescence-like phenotype [28]. In relation to apoptosis, APE1/Ref-1 redox inhibitors have no cytotoxic effect and do not induce apoptosis in a variety of endothelial cell types. In Rf/6a cells and endothelial colony forming cells (ECFCs), APX3330 did not induce apoptosis [30,55]. Furthermore, APX2009 and APX2014 did not induce apoptosis in HRECs in a TUNEL assay [44]. In the treatment of neovascular diseases, this is ideal, since the pre-existing blood vessels should remain intact while proliferative angiogenesis of new blood vessels is halted. 

## 9. Clinical Relevance and Significance

Taking all this evidence together, targeting APE1/Ref-1 redox function may be a new and promising therapeutic approach for neovascular ocular diseases (Table 1). Recently, we showed, for the first time, that systemic and gavage administration of APE1/Ref-1 redox inhibitors reduced L-CNV [44] (Figure 3). With current treatments for neovascular eye diseases consisting of IVT injections, an orally bioavailable drug may have many benefits that outweigh those of IVT injections. For example, a twice-daily pill of an APE1/Ref-1 redox inhibitor would allow for more steady drug levels than those obtained with a monthly injection.

Several studies have been conducted to assess the systemic exposure and potential toxicity in response to APE1/Ref-1 redox inhibitors. APX3330 displayed appropriate pharmacokinetic characteristics in mice, including half-life, area under the curve (AUC) and bioavailability, with no evidence of toxicity or lethality in vivo as determined by weight loss and bone marrow cellularity [40]. A physiological-based pharmacokinetic (PBPK) model was used to predict plasma concentrations and retinal exposure of oral APX3330 and confirmed the pharmacodynamic appropriateness of a 300 mg dose of a twice-daily pill of APX3330 [157]. In addition, the analysis of the safety and profile of APX3330 revealed that APX3330 is safe for chronic dosing from 240 to 600 mg/day. These data support previous clinical findings and pharmacodynamic studies indicating an APX3330-mediated effect upon cancer cells [62,81]. In humans, the dose-limiting toxicity at 720 mg/day was characterized by a spontaneous, reversible, pruritic, diffuse rash [15]. 

In addition, in vitro application of APX2009 to dorsal root ganglia and IP injection of APX3330 for IBD resulted in neuronal protection, demonstrating that APE1/Ref-1 inhibitors are not only not systemically toxic, but are also beneficial and protective [45,91]. APX3330 safety and dosing administration has been established by the Eisai pharmaceutical company (Japan) in non-cancer patients (hepatitis) and toxicology studies for phase I and II trials provided strong evidence that APX3330 does not contribute to toxicity [17,158]. In sum, these findings demonstrate that oral administration of APE1/Ref-1 redox inhibitors may effectively reduce neovascularization in the eye without causing systemic or ocular toxicity. A safe, orally bioavailable drug for neovascular eye disease may also help decrease healthcare costs for patients, reduce office visits and increase patient compliance. 

Current pharmacological treatments for neovascular eye diseases include drugs that target the VEGF signaling pathway. However, many patients who receive these agents do not respond well, become resistant to therapy, or may even experience worsened eye disease, indicating that current anti-VEGF treatments may not be sufficient [8]. In addition, transgenic mice with inducible VEGF expression in the RPE do not develop CNV, indicating that VEGF alone is not sufficient for inducing neovascularization [159]. Approaches such as targeting the redox function of APE1/Ref-1 may be the future of neovascular eye disease therapeutics, as the redox activity influences multiple relevant pathways involved in angiogenesis, inflammation, stress response and cell survival (Figure 1E).

APE1/Ref-1 redox inhibitor APX3330 has begun a phase II clinical trial for DR and DME (Clinical Trials Identifier NCT04692688). The ZETA-1 trial will explore the safety and efficacy of APX3330. ZETA-1 is a randomized, placebo-controlled, double-masked study in which 80 subjects will be enrolled, which include those with moderately-severe to severe non-proliferative DR, mild PDR and DME. Patients will receive twice-daily oral tablets to ensure a steady systemic level of APX3330 for effective and safe therapy. If successful, the trial could reduce the burden of anti-VEGF IVT injections by providing the first oral therapy for treatment of DR/DME. Moreover, it would pave the way for testing APE1/Ref-1 inhibition in other neovascular eye diseases such as nvAMD or even ROP. 

## 10. Outstanding Research Questions

Although many studies indicate that APX3330 strictly inhibits APE1/Ref-1 redox activity and does not affect APE1/Ref-1 endonuclease activity, some evidence suggests that very high concentrations of APX3330 inhibit APE1/Ref-1 endonuclease activity. The concentrations used in these studies were greater than 100 µM, therefore of limited biological significance [18]. There was one report of disruption of APE1/Ref-1 interaction with DNA polymerase β through the disruption of the N-terminal end of APE1/Ref-1, which interacts with DNA polymerase β, but no evidence supporting an alteration of APE1/Ref-1 endonuclease activity [54]. In addition, the possibly neuron-specific activating effect of APX3330 on APE1/Ref-1′s endonuclease activity further suggests some interaction between activities. Because of this neuronal protective effect, the role of APX3330 directly or indirectly impacting the endonuclease activity of APE1/Ref-1 is an area of great interest and study. 

Since downstream targets of APE1/Ref-1 include VEGF, questions still remain if APE1/Ref-1 redox inhibition can be beneficial in anti-VEGF-resistant patients. It also remains to be determined what are all the ocular diseases and patient populations for which APE1/Ref-1 redox inhibitors may be used, since APE1/Ref-1 appears to be implicated in a wide variety of disease states. Because of overlapping pathways implicated in numerous ocular diseases, its use might be applicable for multiple diseases. Moreover, alternative delivery routes for APE1/Ref-1 redox inhibitors could be explored depending on the ocular disease being treated. As mentioned earlier, various studies have evaluated the effects of APE1/Ref-1 redox inhibitors by IVT injection, systemic injection and gavage administration in rodents. Safety, efficacy, systemic exposure, toxicity, patient compliance and many other factors must be considered when weighing these options for human disease therapy [126]. In the case of APX3330, in the 11 clinical trials and over 300 patients treated with oral APX3330 in hepatitis and cancer, a strong safety record and minimal side effects were documented, providing a strong argument for the oral route [62,81]. Oral delivery of APX3330 would be disruptive to the retinal vascular disease field as all approved drugs to date are delivered IVT. 

Attempts were previously made to develop a knockout of APE1/Ref-1, but complete loss of APE1/Ref-1 is embryonically lethal, as expected due to its critical role in the DNA BER pathway along with its redox signaling activities. Heterozygous knockout of APE1/Ref-1 in mice resulted in increased cancer progression, decreased survival rates and increased sensitivity to oxidative stress due to the reduction in the DNA repair capacity in these mice [10,65,160]. To better understand the biology and tissue-specific activity of APE1/Ref-1, a conditional knockout of APE1/Ref-1 or knock-in of endonuclease-dead or DNA repair-dead mutants still need to be developed, such as an APE1/Ref-1-floxed mouse and transduction with Cre adenovirus. Currently, CRISPR knock-ins in cancer cell lines are being developed with mutations in the various Cys residues (C65, C93, C99 and double-Cys mutations) in APE1/Ref-1 to further understand exactly how APE1/Ref-1 functions as a redox signaling molecule in cells [161]. A redox-deficient mutant (C65A) mutant of APE1/Ref-1 can be used to bypass lethality of an APE1/Ref-1 knockout and further identify targets of APE1/Ref-1′s redox function, as evidence suggests that the APE1/Ref-1 DNA repair function is critical for survival, but the redox function is not [61]. Viral tools such as shRNA for testing these mutants in vitro have been beneficial in teasing apart the biology of APE1/Ref-1 in various tissue types [45,74]. Various cell types can be transfected with APE1/Ref-1 siRNA to increase the understanding of the mechanism of APE1/Ref-1 in neovascular eye diseases, coupled with modulation of APE1/Ref-1′s activities using lentivirus to overexpress wild-type and mutant APE1/Ref-1 to examine the effects of APE1/Ref-1 on expression of target genes. 

While we have significant information on APE1/Ref-1′s role in ocular angiogenesis and inflammation, additional studies will clearly be informative. For example, examination of APE1/Ref-1′s influence on novel targets and pathways are currently being explored in our laboratories. Furthermore, APE1/Ref-1 redox inhibitors’ full spectrum of effects in ocular angiogenesis is also unknown. Additional efforts are needed to analyze the mechanism of APE1/Ref-1 and its redox inhibitors in neovascular eye disease. APE1/Ref-1′s effects on various transcriptional targets have been well characterized in various cancers, but its role on other transcriptional targets in the retina and choroid have yet to be elucidated. 

## 11. Conclusions

Given the importance of the redox-regulated transcriptional control by APE1/Ref-1 in neovascular eye disease, modulating this function may make APE1/Ref-1 redox inhibitors potential candidates for treating diseases such as nvAMD, PDR/DME and ROP. Because APE1/Ref-1 simultaneously affects angiogenesis, inflammation, stress response and other key pathways implicated in neovascular eye disease, targeting APE1/Ref-1 may be a novel approach that has the potential to improve disease outcomes in many patients and may be a better therapeutic tactic than current anti-VEGF therapies. APE1/Ref-1 redox inhibitors hold much promise for retinal and choroidal neovascularization by targeting multiple pathways and perhaps bypassing issues that surround IVT anti-VEGF therapy, thereby alleviating some of the burden associated with current therapies. 

## Figures and Tables

**Table 1:** Evidence linking APE1/Ref-1 inhibitors with eye disease. Redox inhibitors APX3330, APX2009 and APX2014 have been assessed in multiple systems. For structures of compounds mentioned, refer to Figure 1. Abbreviations: RVECs, retinal vascular endothelial cells; ARPE-19, adult retinal pigment epithelium-19 cell line; CI, cell index; MCP-1, monocyte chemoattractant protein-1; oxLDL, oxidized low density lipoprotein; ROS, reactive oxygen species; VEGF, vascular endothelial growth factor; NF-κB, Nuclear factor κ light-chain-enhancer of activated B cells; Nrf2/Nrf1, Nuclear factor erythroid 2-related factor 2 and 1; HIF-1, hypoxia-inducible factor 1; CBF/NF-Y, CCAAT-binding factor; YY1, Ying yang-1; MTF1, metal regulatory transcription factor 1; HSF-1, heat shock factor 1; Rf-6a, macaque choroidal endothelial cell-like cell line; STAT3, signal transducer and activator of transcription 3; HRECs, human retinal microvascular endothelial cells; *VCAM1*, vascular cell adhesion molecule 1; *CCL20*, C-C motif chemokine ligand 20; *VEGFA*, vascular endothelial growth factor A; TUNEL, Terminal deoxynucleotidyl transferase dUTP nick end labeling; IVT, intravitreal; L-CNV, laser-induced choroidal neovascularization; IP, intraperitoneal.

Intervention	System	Findings	Reference
In vitro			
APX3330	RVECs	Dose-dependently suppressed proliferation, migration and tube formation	[30]
APX3330	RVECs	Reduced CI	[30]
APX3330	ARPE-19	Prevented apoptosis and reduced the upsurge of MCP-1 following induction of pathological stress with oxLDL	[28]
APX3330	ARPE-19	Reduced accumulation of intracellular ROS, secretion of VEGF and effectively blocked the upsurge of NF-κB in response to induced pathological stress by oxLDL	[28]
APX3330	ARPE-19	Protected the cells from a stress-induced senescence-like phenotype	[28]
APX3330	ARPE-19	Decreased the transcription activities of Nrf2/Nrf1, p53, NF-κB, HIF-1, CBF/NF-Y, YY1, MTF1 and HSF-1	[28]
APX3330	Rf/6a	Reduced p65 expression and NF-κB transcriptional activity	[55]
APX3330	Rf/6a	Dose-dependently downregulated the production of MCP-1	[55]
APX3330	Rf/6a	Reduced STAT3 and NF-κB DNA binding activity	[55]
APX3330	Rf/6a	Dose-dependently suppressed angiogenesis (proliferation, migration and tube formation)	[55]
APX3330	Rf/6a	Did not induce apoptosis	[55]
APX3330 and Bevacizumab (antibody)	Rf/6a	Additive decline in migration, tube formation and proliferation	[55]
APX2009APX2014	HRECs; Rf/6a	Dose-dependently decreased choroidal sprouting, proliferation, tube formation and endothelial cell migration	[44]
APX2009APX2014	HRECs	Dose-dependently reduced translocation of the p65 subunit of NF-κB into the nucleus and decreased downstream mRNA targets of NF-κB including *VCAM1, CCL20* and *VEGFA*	[44]
APX2009APX2014	HRECs	Did not induce apoptosis in a TUNEL assay and blocked cells from entering the S phase	[44]
In vivo			
APX3330	*Vldlr^−/−^* mice	Single IVT injection of 20 μM decreased neovascularization	[30]
APX3330	L-CNV mice	Single IVT injection for final intraocular concentration of 20 μM suppressed L-CNV lesion area	[28,55]
APX3330	L-CNV mice	IP injection twice a day at 50 mg/kg for 5 days on and 2 days off for two weeks reduced L-CNV volume by 25%	[44]
APX2009	L-CNV mice	IP injection (25 mg/kg twice daily for two weeks) decreased L-CNV volume without causing systemic toxicity	[44]
APX3330	L-CNV mice	Gavage administration of either 25 mg/kg or 50 mg/kg gavage of APX3330 twice daily for 14 days resulted in decrease of lesion size by >50%	Figure 3

## Data Availability

The data presented in this study are available on request from the corresponding author.

## Abbreviations

AD	Alzheimer’s disease
ALS	Amyotrophic lateral sclerosis
AMD	Age-related macular degeneration
AP-1	Activator protein 1
APE1/Ref-1	Apurinic/apyrimidinic endonuclease 1/reduction-oxidation factor 1
APX2009	(*E*)-*N,N*-diethyl-2-((3-methoxy-1,4-dihydronaphthalen-2-yl)methylene)pentanamide
APX2014	(*E*)-*N*-methoxy-2-((3-methoxy-1,4-dioxo-1,4-dihydronaphthalen-2-yl)methylene)pentanamide
APX3330	(2*E*)-2-[(4,5-dimethoxy-2-methyl-3,6-dioxo-1,4-cyclohexadien-1-yl)methyelene]-undecanoic acid
ARE	Antioxidant response element
ARPE-19	Adult retinal pigment epithelium-19
AUC	Area under the curve
BER	Base-excision repair
BMI	Body mass index
CA9	Carbonic anhydrase 9
CBF/NF-Y	CCAAT-binding factor
CCL20	C-C motif chemokine ligand 20
CI	Cell index
CIPN	Chemotherapy-induced peripheral neuropathy
CNS	Central nervous system
CREB	cAMP-responsive element (CRE)-binding protein
CXCR4	CXC motif chemokine receptor 4
DME	Diabetic macular edema
DR	Diabetic retinopathy
ECFCs	Erythroid-colony-forming unit cells
ECM	Extracellular matrix
EMSA	Electrophoretic mobility shift assay
ERK	Extracellular-signal-regulated kinase
HEY-C2	Ovarian cancer cell line
HIF-1	Hypoxia-inducible factor 1
HIF-1α	Hypoxia-inducible factor 1 α subunit
HIF-1β	Hypoxia-inducible factor 1 β subunit
HO-1	Heme oxygenase 1
HRECs	Human retinal microvascular endothelial cells
HSF-1	Heat shock factor 1
HUVEC	Human umbilical vein endothelial cells
ICAM-1	Intracellular adhesion molecule 1
IBD	Inflammatory bowel disease
IKK	IκB kinase
IL-1β	Interleukin 1β
IL-6	Interleukin 6
IL-8	Interleukin 8
IP	Intraperitoneal
IVT	Intravitreal
JAK/STAT	Janus kinase/signal transducer and activator of transcription
LC-MS/MS	Liquid chromatography with tandem mass spectrometry
L-CNV	Laser-induced choroidal neovascularization
MCP-1	Monocyte chemoattractant protein-1
MPP+	1-methyl-4-phenylpyridinium
mtDNA	Mitochondrial DNA
MTF-1	Metal regulatory transcription factor 1
NF-κB	Nuclear factor κ light-chain-enhancer of activated B cells
NPDR	Non-proliferative diabetic retinopathy
NPM1	Nucleophosmin 1
Nrf1	Nuclear factor erythroid 2-related factor 1
Nrf2	Nuclear factor erythroid 2-related factor 2
nvAMD	Neovascular age-related macular degeneration
OCT	Optical coherence tomography
oxLDL	Oxidized low density lipoprotein
PBPK	Physiological-based pharmacokinetic
PD	Parkinson’s disease
PDAC	Pancreatic ductal adenocarcinoma
PDGF-B	Platelet-derived growth factor subunit B
PDR	Proliferative diabetic retinopathy
PI3K/Akt	Phosphoinositide-3-kinase
PEBP-2	Polyomavirus enhancer-binding protein 2
Rf/6a	Macaque choroidal endothelial cell-like cell line
ROP	Retinopathy of prematurity
ROS	Reactive oxygen species
RPE	Retinal pigment epithelium
RVECs	Retinal vascular endothelial cells
SKOV-3X	Human ovarian cancer cell line
STAT3	Signal transducer and activator of transcription 3
TF(s)	Transcription factor(s)
TNF-α	Tumor necrosis factor alpha
TUNEL	Terminal deoxynucleotidyl transferase dUTP nick end labeling
VEGF	Vascular endothelial growth factor
VE-PTP	Vascular endothelial protein tyrosine phosphatase
VHL	Von Hippel–Lindau
YY1	Ying yang-1

## References

[1] Cabral T., Mello L.G.M., Lima L.H., Polido J., Regatieri C.V., Belfort R., Mahajan V.B. (2017). Retinal and choroidal angiogenesis: A review of new targets. Int. J. Retin. Vitr..

[2] Lee P., Wang C.C., Adamis A.P. (1998). Ocular neovascularization: An epidemiologic review. Surv. Ophthalmol..

[3] Dreyfuss J.L., Giordano R.J., Regatieri C.V. (2015). Ocular angiogenesis. J. Ophthalmol..

[4] Wong W.L., Su X., Li X., Cheung C.M., Klein R., Cheng C.Y., Wong T.Y. (2014). Global prevalence of age-related macular degeneration and disease burden projection for 2020 and 2040: A systematic review and meta-analysis. Lancet Glob. Health.

[5] Zheng Y., He M., Congdon N. (2012). The worldwide epidemic of diabetic retinopathy. Indian J. Ophthalmol..

[6] Blencowe H., Lawn J.E., Vazquez T., Fielder A., Gilbert C. (2013). Preterm-associated visual impairment and estimates of retinopathy of prematurity at regional and global levels for 2010. Pediatr. Res..

[7] Solebo A.L., Teoh L., Rahi J. (2017). Epidemiology of blindness in children. Arch. Dis Child..

[8] Yang S., Zhao J., Sun X. (2016). Resistance to anti-VEGF therapy in neovascular age-related macular degeneration: A comprehensive review. Drug Des. Devel. Ther..

[9] Kelley M.R., Georgiadis M.M., Fishel M.L. (2012). APE1/Ref-1 role in redox signaling: Translational applications of targeting the redox function of the DNA repair/redox protein APE1/Ref-1. Curr. Mol. Pharmacol..

[10] Li M., Wilson D.M. (2014). Human apurinic/apyrimidinic endonuclease 1. Antioxid. Redox Signal..

[11] Kane C.M., Linn S. (1981). Purification and characterization of an apurinic/apyrimidinic endonuclease from HeLa cells. J. Biol. Chem..

[12] Grafstrom R.H., Shaper N.L., Grossman L. (1982). Human placental apurinic/apyrimidinic endonuclease. Mechanism of action. J. Biol. Chem..

[13] Shaper N.L., Grafstrom R.H., Grossman L. (1982). Human placental apurinic/apyrimidinic endonuclease. Its isolation and characterization. J. Biol. Chem..

[14] Xanthoudakis S., Curran T. (1992). Identification and characterization of Ref-1, a nuclear protein that facilitates AP-1 DNA-binding activity. EMBO J..

[15] Caston R.A., Gampala S., Armstrong L., Messmann R.A., Fishel M.L., Kelley M.R. (2021). The multifunctional APE1 DNA repair-redox signaling protein as a drug target in human disease. Drug Discov. Today.

[16] Georgiadis M.M., Luo M., Gaur R.K., Delaplane S., Li X., Kelley M.R. (2008). Evolution of the redox function in mammalian apurinic/apyrimidinic endonuclease. Mutat. Res..

[17] Shah F., Logsdon D., Messmann R.A., Fehrenbacher J.C., Fishel M.L., Kelley M.R. (2017). Exploiting the Ref-1-APE1 node in cancer signaling and other diseases: From bench to clinic. NPJ Precis. Oncol..

[18] Zhang J., Luo M., Marasco D., Logsdon D., LaFavers K.A., Chen Q., Reed A., Kelley M.R., Gross M.L., Georgiadis M.M. (2013). Inhibition of apurinic/apyrimidinic endonuclease I’s redox activity revisited. Biochemistry.

[19] Gorman M.A., Morera S., Rothwell D.G., de La Fortelle E., Mol C.D., Tainer J.A., Hickson I.D., Freemont P.S. (1997). The crystal structure of the human DNA repair endonuclease HAP1 suggests the recognition of extra-helical deoxyribose at DNA abasic sites. EMBO J..

[20] Xanthoudakis S., Miao G.G., Curran T. (1994). The redox and DNA-repair activities of Ref-1 are encoded by nonoverlapping domains. Proc. Natl. Acad. Sci. USA.

[21] Evans A.R., Limp-Foster M., Kelley M.R. (2000). Going APE over ref-1. Mutat Res..

[22] Izumi T., Mitra S. (1998). Deletion analysis of human AP-endonuclease: Minimum sequence required for the endonuclease activity. Carcinogenesis.

[23] Vascotto C., Fantini D., Romanello M., Cesaratto L., Deganuto M., Leonardi A., Radicella J.P., Kelley M.R., D’Ambrosio C., Scaloni A. (2009). APE1/Ref-1 interacts with NPM1 within nucleoli and plays a role in the rRNA quality control process. Mol. Cell Biol..

[24] He H., Chen Q., Georgiadis M.M. (2014). High-resolution crystal structures reveal plasticity in the metal binding site of apurinic/apyrimidinic endonuclease I. Biochemistry.

[25] Sehnal D., Bittrich S., Deshpande M., Svobodová R., Berka K., Bazgier V., Velankar S., Burley S.K., Koča J., Rose A.S. (2021). Mol* Viewer: Modern web app for 3D visualization and analysis of large biomolecular structures. Nucleic Acid. Res..

[26] Luo M., Zhang J., He H., Su D., Chen Q., Gross M.L., Kelley M.R., Georgiadis M.M. (2012). Characterization of the redox activity and disulfide bond formation in apurinic/apyrimidinic endonuclease. Biochemistry.

[27] Lee Y.R., Joo H.K., Jeon B.H. (2020). The biological role of apurinic/apyrimidinic endonuclease1/redox factor-1 as a therapeutic target for vascular inflammation and as a serologic biomarker. Biomedicines.

[28] Li Y., Liu X., Zhou T., Kelley M.R., Edwards P., Gao H., Qiao X. (2014). Inhibition of APE1/Ref-1 redox activity rescues human retinal pigment epithelial cells from oxidative stress and reduces choroidal neovascularization. Redox Biol..

[29] Chiarini L.B., Freitas F.G., Petrs-Silva H., Linden R. (2000). Evidence that the bifunctional redox factor / AP endonuclease Ref-1 is an anti-apoptotic protein associated with differentiation in the developing retina. Cell Death Differ..

[30] Jiang A., Gao H., Kelley M.R., Qiao X. (2011). Inhibition of APE1/Ref-1 redox activity with APX3330 blocks retinal angiogenesis in vitro and in vivo. Vis. Res..

[31] Lee Y.R., Joo H.K., Lee E.O., Park M.S., Cho H.S., Kim S., Jin H., Jeong J.O., Kim C.S., Jeon B.H. (2020). Plasma APE1/Ref-1 correlates with atherosclerotic inflammation in ApoE(−/−) mice. Biomedicines.

[32] Jin S.A., Lim B.K., Seo H.J., Kim S.K., Ahn K.T., Jeon B.H., Jeong J.O. (2017). Elevation of serum APE1/Ref-1 in experimental murine myocarditis. Int. J. Mol. Sci..

[33] Lee Y.R., Joo H.K., Lee E.O., Cho H.S., Choi S., Kim C.S., Jeon B.H. (2019). ATP binding cassette transporter A1 is involved in extracellular secretion of acetylated APE1/Ref-1. Int. J. Mol. Sci..

[34] Eads J.R., Krishnamurthi S.S., Saltzman J., Bokar J.A., Savvides P., Meropol N.J., Gibbons J., Koon H., Sharma N., Rogers L. (2021). Phase I clinical trial of temozolomide and methoxyamine (TRC-102), an inhibitor of base excision repair, in patients with advanced solid tumors. Invest. New Drugs.

[35] Poletto M., Malfatti M.C., Dorjsuren D., Scognamiglio P.L., Marasco D., Vascotto C., Jadhav A., Maloney D.J., Wilson D.M., Simeonov A. (2016). Inhibitors of the apurinic/apyrimidinic endonuclease 1 (APE1)/nucleophosmin (NPM1) interaction that display anti-tumor properties. Mol. Carcinog..

[36] Choi S., Joo H.K., Jeon B.H. (2016). Dynamic regulation of APE1/Ref-1 as a therapeutic target protein. Chonnam. Med. J..

[37] Cardoso A.A., Jiang Y., Luo M., Reed A.M., Shahda S., He Y., Maitra A., Kelley M.R., Fishel M.L. (2012). APE1/Ref-1 regulates STAT3 transcriptional activity and APE1/Ref-1-STAT3 dual-targeting effectively inhibits pancreatic cancer cell survival. PLoS ONE.

[38] Fishel M.L., Colvin E.S., Luo M., Kelley M.R., Robertson K.A. (2010). Inhibition of the redox function of APE1/Ref-1 in myeloid leukemia cell lines results in a hypersensitive response to retinoic acid-induced differentiation and apoptosis. Exp. Hematol..

[39] Fishel M.L., Wu X., Devlin C.M., Logsdon D.P., Jiang Y., Luo M., He Y., Yu Z., Tong Y., Lipking K.P. (2015). Apurinic/apyrimidinic endonuclease/redox factor-1 (APE1/Ref-1) redox function negatively regulates NRF2. J. Biol. Chem..

[40] Fishel M.L., Jiang Y., Rajeshkumar N.V., Scandura G., Sinn A.L., He Y., Shen C., Jones D.R., Pollok K.E., Ivan M. (2011). Impact of APE1/Ref-1 redox inhibition on pancreatic tumor growth. Mol. Cancer Ther..

[41] Su D., Delaplane S., Luo M., Rempel D.L., Vu B., Kelley M.R., Gross M.L., Georgiadis M.M. (2011). Interactions of apurinic/apyrimidinic endonuclease with a redox inhibitor: Evidence for an alternate conformation of the enzyme. Biochemistry.

[42] Luo M., Delaplane S., Jiang A., Reed A., He Y., Fishel M., Nyland R.L., Borch R.F., Qiao X., Georgiadis M.M. (2008). Role of the multifunctional DNA repair and redox signaling protein Ape1/Ref-1 in cancer and endothelial cells: Small-molecule inhibition of the redox function of Ape1. Antioxid. Redox Signal..

[43] Jedinak A., Dudhgaonkar S., Kelley M.R., Sliva D. (2011). Apurinic/apyrimidinic endonuclease 1 regulates inflammatory response in macrophages. Anticancer Res..

[44] Sardar Pasha S.P.B., Sishtla K., Sulaiman R.S., Park B., Shetty T., Shah F., Fishel M.L., Wikel J.H., Kelley M.R., Corson T.W. (2018). Ref-1/APE1 inhibition with novel small molecules blocks ocular neovascularization. J. Pharmacol. Exp. Ther..

[45] Kelley M.R., Wikel J.H., Guo C., Pollok K.E., Bailey B.J., Wireman R., Fishel M.L., Vasko M.R. (2016). Identification and characterization of new chemical entities targeting apurinic/apyrimidinic endonuclease 1 for the prevention of chemotherapy-induced peripheral neuropathy. J. Pharmacol. Exp. Ther..

[46] Kelley M.R., Luo M., Reed A., Su D., Delaplane S., Borch R.F., Nyland R.L., Gross M.L., Georgiadis M.M. (2011). Functional analysis of novel analogues of E3330 that block the redox signaling activity of the multifunctional AP endonuclease/redox signaling enzyme APE1/Ref-1. Antioxid. Redox Signal..

[47] Laev S.S., Salakhutdinov N.F., Lavrik O.I. (2017). Inhibitors of nuclease and redox activity of apurinic/apyrimidinic endonuclease 1/redox effector factor 1 (APE1/Ref-1). Bioorg. Med. Chem..

[48] Nelson K.M., Dahlin J.L., Bisson J., Graham J., Pauli G.F., Walters M.A. (2017). The essential medicinal chemistry of curcumin. J. Med. Chem..

[49] Nyland R.L., Luo M., Kelley M.R., Borch R.F. (2010). Design and synthesis of novel quinone inhibitors targeted to the redox function of apurinic/apyrimidinic endonuclease 1/redox enhancing factor-1 (Ape1/ref-1). J. Med. Chem..

[50] Logsdon D.P., Shah F., Carta F., Supuran C.T., Kamocka M., Jacobsen M.H., Sandusky G.E., Kelley M.R., Fishel M.L. (2018). Blocking HIF signaling via novel inhibitors of CA9 and APE1/Ref-1 dramatically affects pancreatic cancer cell survival. Sci. Rep..

[51] Gampala S., Shah F., Lu X., Moon H.R., Babb O., Umesh Ganesh N., Sandusky G., Hulsey E., Armstrong L., Mosley A.L. (2021). Ref-1 redox activity alters cancer cell metabolism in pancreatic cancer: Exploiting this novel finding as a potential target. J. Exp. Clin. Cancer Res..

[52] Hiramoto M., Shimizu N., Sugimoto K., Tang J., Kawakami Y., Ito M., Aizawa S., Tanaka H., Makino I., Handa H. (1998). Nuclear targeted suppression of NF-kappa B activity by the novel quinone derivative E3330. J. Immunol..

[53] Cai Z., Kotzin J.J., Ramdas B., Chen S., Nelanuthala S., Palam L.R., Pandey R., Mali R.S., Liu Y., Kelley M.R. (2018). Inhibition of inflammatory signaling in Tet2 mutant preleukemic cells mitigates stress-induced abnormalities and clonal hematopoiesis. Cell Stem Cell.

[54] Li M., Dai N., Wang D., Zhong Z. (2019). Distinct APE1 activities affect the regulation of VEGF transcription under hypoxic conditions. Comput. Struct. Biotechnol. J..

[55] Li Y., Liu X., Zhou T., Kelley M.R., Edwards P.A., Gao H., Qiao X. (2014). Suppression of choroidal neovascularization through inhibition of APE1/Ref-1 redox activity. Invest. Ophthalmol. Vis. Sci..

[56] Shah F., Goossens E., Atallah N.M., Grimard M., Kelley M.R., Fishel M.L. (2017). APE1/Ref-1 knockdown in pancreatic ductal adenocarcinoma—Characterizing gene expression changes and identifying novel pathways using single-cell RNA sequencing. Mol. Oncol..

[57] McIlwain D.W., Fishel M.L., Boos A., Kelley M.R., Jerde T.J. (2018). APE1/Ref-1 redox-specific inhibition decreases survivin protein levels and induces cell cycle arrest in prostate cancer cells. Oncotarget.

[58] Tao Y., Poornima V., Michael C., Alex Z., Peng Y., Ruizhuo N., Xiaoxi Q., Kelley M.R., Jieli C. (2018). APX3330 promotes neurorestorative effects after stroke in type one diabetic rats. Aging Dis..

[59] Bhat A.A., Lu H., Soutto M., Capobianco A., Rai P., Zaika A., El-Rifai W. (2018). Exposure of Barrett’s and esophageal adenocarcinoma cells to bile acids activates EGFR-STAT3 signaling axis via induction of APE1. Oncogene.

[60] Cesaratto L., Codarin E., Vascotto C., Leonardi A., Kelley M.R., Tiribelli C., Tell G. (2013). Specific inhibition of the redox activity of APE1/Ref-1 by E3330 blocks TNF-alpha-induced activation of IL-8 production in liver cancer cell lines. PLoS ONE.

[61] Sriramajayam K., Peng D., Lu H., Zhou S., Bhat N., McDonald O.G., Que J., Zaika A., El-Rifai W. (2021). Activation of NRF2 by APE1/REF1 is redox-dependent in Barrett’s related esophageal adenocarcinoma cells. Redox Biol..

[62] Kelley M.R., Shahda S., Lakhani N.J., O’Neil B., Chu L., Anderson A.K., Wan J., Mosley A.L., Liu H., Messmann R.A. A phase I study targeting the APE1/Ref-1 DNA repair-redox signaling protein with the APX3330 inhibitor [abstract]. Proceedings of the AACR-NCI-EORTC International Conference on Molecular Targets and Cancer Therapeutics.

[63] Shimizu N., Sugimoto K., Tang J., Nishi T., Sato I., Hiramoto M., Aizawa S., Hatakeyama M., Ohba R., Hatori H. (2000). High-performance affinity beads for identifying drug receptors. Nat. Biotechnol..

[64] Codrich M., Comelli M., Malfatti M.C., Mio C., Ayyildiz D., Zhang C., Kelley M.R., Terrosu G., Pucillo C.E.M., Tell G. (2019). Inhibition of APE1-endonuclease activity affects cell metabolism in colon cancer cells via a p53-dependent pathway. DNA Repair.

[65] Bhakat K.K., Mantha A.K., Mitra S. (2009). Transcriptional regulatory functions of mammalian AP-endonuclease (APE1/Ref-1), an essential multifunctional protein. Antioxid. Redox Signal..

[66] Bobola M.S., Blank A., Berger M.S., Stevens B.A., Silber J.R. (2001). Apurinic/apyrimidinic endonuclease activity is elevated in human adult gliomas. Clin. Cancer Res..

[67] Carrero P., Okamoto K., Coumailleau P., O’Brien S., Tanaka H., Poellinger L. (2000). Redox-regulated recruitment of the transcriptional coactivators CREB-binding protein and SRC-1 to hypoxia-inducible factor 1alpha. Mol. Cell Biol..

[68] Kelley M.R., Cheng L., Foster R., Tritt R., Jiang J., Broshears J., Koch M. (2001). Elevated and altered expression of the multifunctional DNA base excision repair and redox enzyme Ape1/Ref-1 in prostate cancer. Clin. Cancer Res..

[69] Koukourakis M.I., Giatromanolaki A., Kakolyris S., Sivridis E., Georgoulias V., Funtzilas G., Hickson I.D., Gatter K.C., Harris A.L. (2001). Nuclear expression of human apurinic/apyrimidinic endonuclease (HAP1/Ref-1) in head-and-neck cancer is associated with resistance to chemoradiotherapy and poor outcome. Int. J. Radiat. Oncol. Biol. Phys..

[70] Puglisi F., Aprile G., Minisini A.M., Barbone F., Cataldi P., Tell G., Kelley M.R., Damante G., Beltrami C.A., Di Loreto C. (2001). Prognostic significance of Ape1/Ref-1 subcellular localization in non-small cell lung carcinomas. Anticancer Res..

[71] Robertson K.A., Bullock H.A., Xu Y., Tritt R., Zimmerman E., Ulbright T.M., Foster R.S., Einhorn L.H., Kelley M.R. (2001). Altered expression of Ape1/Ref-1 in germ cell tumors and overexpression in NT2 cells confers resistance to bleomycin and radiation. Cancer Res..

[72] Fishel M.L., Kelley M.R. (2007). The DNA base excision repair protein Ape1/Ref-1 as a therapeutic and chemopreventive target. Mol. Aspects Med..

[73] Kelley M.R., Logsdon D., Fishel M.L. (2014). Targeting DNA repair pathways for cancer treatment: What’s new?. Future Oncol..

[74] Logsdon D.P., Grimard M., Luo M., Shahda S., Jiang Y., Tong Y., Yu Z., Zyromski N., Schipani E., Carta F. (2016). Regulation of HIF1alpha under hypoxia by APE1/Ref-1 impacts CA9 expression: Dual targeting in patient-derived 3D pancreatic cancer models. Mol. Cancer Ther..

[75] Jiang Y., Zhou S., Sandusky G.E., Kelley M.R., Fishel M.L. (2010). Reduced expression of DNA repair and redox signaling protein APE1/Ref-1 impairs human pancreatic cancer cell survival, proliferation, and cell cycle progression. Cancer Invest..

[76] Sharbeen G., McCarroll J., Goldstein D., Phillips P.A. (2015). Exploiting base excision repair to improve therapeutic approaches for pancreatic cancer. Front. Nutr..

[77] Gampala S., Shah F., Zhang C., Rhodes S.D., Babb O., Grimard M., Wireman R.S., Rad E., Calver B., Bai R.Y. (2021). Exploring transcriptional regulators Ref-1 and STAT3 as therapeutic targets in malignant peripheral nerve sheath tumours. Br. J. Cancer.

[78] Heisel C., Yousif J., Mijiti M., Charizanis K., Brigell M., Corson T.W., Kelley M.R. (2021). APE1/Ref-1 as a novel target for retinal diseases. Cell. Signal..

[79] Zou G.M., Karikari C., Kabe Y., Handa H., Anders R.A., Maitra A. (2009). The Ape-1/Ref-1 redox antagonist E3330 inhibits the growth of tumor endothelium and endothelial progenitor cells: Therapeutic implications in tumor angiogenesis. J. Cell Physiol..

[80] Lee Y.R., Park M.S., Joo H.K., Kim K.M., Kim J., Jeon B.H., Choi S. (2018). Therapeutic positioning of secretory acetylated APE1/Ref-1 requirement for suppression of tumor growth in triple-negative breast cancer in vivo. Sci. Rep..

[81] Shahda S., Lakhani N.J., O’Neil B., Rasco D.W., Wan J., Mosley A.L., Liu H., Kelley M.R., Messmann R.A. (2019). A phase I study of the APE1 protein inhibitor APX3330 in patients with advanced solid tumors. J. Clin. Oncol..

[82] Davydov V., Hansen L.A., Shackelford D.A. (2003). Is DNA repair compromised in Alzheimer’s disease?. Neurobiol. Aging.

[83] Marcon G., Tell G., Perrone L., Garbelli R., Quadrifoglio F., Tagliavini F., Giaccone G. (2009). APE1/Ref-1 in Alzheimer’s disease: An immunohistochemical study. Neurosci. Lett..

[84] Tan Z., Sun N., Schreiber S.S. (1998). Immunohistochemical localization of redox factor-1 (Ref-1) in Alzheimer’s hippocampus. Neuroreport.

[85] Shaikh A.Y., Martin L.J. (2002). DNA base-excision repair enzyme apurinic/apyrimidinic endonuclease/redox factor-1 is increased and competent in the brain and spinal cord of individuals with amyotrophic lateral sclerosis. Neuromol. Med..

[86] Kang B., Mu S., Yang Q., Guo S., Chen X., Guo H. (2017). Ape1 protects against MPP+-induced neurotoxicity through ERK1/2 signaling in PC12 cells. Neuroreport.

[87] Leak R.K., Li P., Zhang F., Sulaiman H.H., Weng Z., Wang G., Stetler R.A., Shi Y., Cao G., Gao Y. (2015). Apurinic/apyrimidinic endonuclease 1 upregulation reduces oxidative DNA damage and protects hippocampal neurons from ischemic injury. Antioxid. Redox Signal..

[88] Stetler R.A., Gao Y., Leak R.K., Weng Z., Shi Y., Zhang L., Pu H., Zhang F., Hu X., Hassan S. (2016). APE1/Ref-1 facilitates recovery of gray and white matter and neurological function after mild stroke injury. Proc. Natl. Acad. Sci. USA.

[89] Kelley M.R., Jiang Y., Guo C., Reed A., Meng H., Vasko M.R. (2014). Role of the DNA base excision repair protein, APE1 in cisplatin, oxaliplatin, or carboplatin induced sensory neuropathy. PLoS ONE.

[90] Mijiti M., Caston R., Gampala S., Fishel M.L., Fehrenbacher J.C., Kelley M.R. (2021). APE1/Ref-1—One target with multiple indications: Emerging aspects and new directions. J. Cell. Signal..

[91] Sahakian L., Filippone R.T., Stavely R., Robinson A.M., Yan X.S., Abalo R., Eri R., Bornstein J.C., Kelley M.R., Nurgali K. (2021). Inhibition of APE1/Ref-1 redox signaling alleviates intestinal dysfunction and damage to myenteric neurons in a mouse model of spontaneous chronic colitis. Inflamm. Bowel Dis..

[92] Campochiaro P.A. (2013). Ocular neovascularization. J. Mol. Med..

[93] Bhutto I., Lutty G. (2012). Understanding age-related macular degeneration (AMD): Relationships between the photoreceptor/retinal pigment epithelium/Bruch’s membrane/choriocapillaris complex. Mol. Aspects Med..

[94] Klein R., Cruickshanks K.J., Myers C.E., Sivakumaran T.A., Iyengar S.K., Meuer S.M., Schubert C.R., Gangnon R.E., Klein B.E. (2013). The relationship of atherosclerosis to the 10-year cumulative incidence of age-related macular degeneration: The Beaver Dam studies. Ophthalmology.

[95] Pennington K.L., DeAngelis M.M. (2016). Epidemiology of age-related macular degeneration (AMD): Associations with cardiovascular disease phenotypes and lipid factors. Eye Vis..

[96] Fleckenstein M., Keenan T.D.L., Guymer R.H., Chakravarthy U., Schmitz-Valckenberg S., Klaver C.C., Wong W.T., Chew E.Y. (2021). Age-related macular degeneration. Nat. Rev. Dis. Primers.

[97] Edwards M., Lutty G.A. (2021). Bruch’s membrane and the choroid in age-related macular degeneration. Adv. Exp. Med. Biol..

[98] Spaide R.F., Jaffe G.J., Sarraf D., Freund K.B., Sadda S.R., Staurenghi G., Waheed N.K., Chakravarthy U., Rosenfeld P.J., Holz F.G. (2020). Consensus nomenclature for reporting neovascular age-related macular degeneration data: Consensus on neovascular age-related macular degeneration nomenclature study group. Ophthalmology.

[99] Seddon J.M. (2017). Macular degeneration epidemiology: Nature-nurture, lifestyle factors, genetic risk, and gene-environment interactions—The Weisenfeld Award Lecture. Invest. Ophthalmol. Vis. Sci..

[100] Ung C., Lains I., Miller J.W., Kim I.K. (2021). Current management of age-related macular degeneration. Adv. Exp. Med. Biol..

[101] Klein B.E. (2007). Overview of epidemiologic studies of diabetic retinopathy. Ophthalmic. Epidemiol..

[102] Antonetti D.A., Klein R., Gardner T.W. (2012). Diabetic retinopathy. N. Engl. J. Med..

[103] Peet D.J., Kittipassorn T., Wood J.P., Chidlow G., Casson R.J. (2017). HIF signalling: The eyes have it. Exp. Cell Res..

[104] Hendrick A.M., Gibson M.V., Kulshreshtha A. (2015). Diabetic retinopathy. Prim. Care.

[105] Capitao M., Soares R. (2016). Angiogenesis and inflammation crosstalk in diabetic retinopathy. J. Cell Biochem..

[106] Simo R., Sundstrom J.M., Antonetti D.A. (2014). Ocular anti-VEGF therapy for diabetic retinopathy: The role of VEGF in the pathogenesis of diabetic retinopathy. Diabetes Care.

[107] Nguyen Q.D., Shah S.M., Khwaja A.A., Channa R., Hatef E., Do D.V., Boyer D., Heier J.S., Abraham P., Thach A.B. (2010). Two-year outcomes of the ranibizumab for edema of the macula in diabetes (READ-2) study. Ophthalmology.

[108] Michaelides M., Kaines A., Hamilton R.D., Fraser-Bell S., Rajendram R., Quhill F., Boos C.J., Xing W., Egan C., Peto T. (2010). A prospective randomized trial of intravitreal bevacizumab or laser therapy in the management of diabetic macular edema (BOLT study) 12-month data: Report 2. Ophthalmology.

[109] Wallsh J.O., Gallemore R.P. (2021). Anti-VEGF-resistant retinal diseases: A review of the latest treatment options. Cells.

[110] Ludwig C.A., Chen T.A., Hernandez-Boussard T., Moshfeghi A.A., Moshfeghi D.M. (2017). The epidemiology of retinopathy of prematurity in the United States. Ophthalmic. Surg. Lasers Imaging Retin..

[111] Good W.V., Hardy R.J., Dobson V., Palmer E.A., Phelps D.L., Quintos M., Tung B. (2005). Early Treatment for Retinopathy of Prematurity Cooperative Group, The incidence and course of retinopathy of prematurity: Findings from the Early Treatment for Retinopathy of Prematurity Study. Pediatrics.

[112] Gupta V.P., Dhaliwal U., Sharma R., Gupta P., Rohatgi J. (2004). Retinopathy of prematurity—Risk factors. Indian J. Pediatr..

[113] Smith L.E. (2003). Pathogenesis of retinopathy of prematurity. Semin. Neonatol..

[114] Chen J., Stahl A., Hellstrom A., Smith L.E. (2011). Current update on retinopathy of prematurity: Screening and treatment. Curr. Opin. Pediatr..

[115] Wallace D.K., Dean T.W., Hartnett M.E., Kong L., Smith L.E., Hubbard G.B., McGregor M.L., Jordan C.O., Mantagos I.S., Bell E.F. (2018). A dosing study of bevacizumab for retinopathy of prematurity: Late recurrences and additional treatments. Ophthalmology.

[116] Wallace D.K., Kraker R.T., Freedman S.F., Crouch E.R., Bhatt A.R., Hartnett M.E., Yang M.B., Rogers D.L., Hutchinson A.K., VanderVeen D.K. (2020). Short-term outcomes after very low-dose intravitreous bevacizumab for retinopathy of prematurity. JAMA Ophthalmol..

[117] Cornel S., Adriana I.D., Mihaela T.C., Speranta S., Algerino S., Mehdi B., Jalaladin H.R. (2015). Anti-vascular endothelial growth factor indications in ocular disease. Rom. J. Ophthalmol..

[118] Ammar M.J., Hsu J., Chiang A., Ho A.C., Regillo C.D. (2020). Age-related macular degeneration therapy: A review. Curr. Opin. Ophthalmol..

[119] Bloch S.B., Larsen M., Munch I.C. (2012). Incidence of legal blindness from age-related macular degeneration in Denmark: Year 2000 to 2010. Am. J. Ophthalmol..

[120] Avery R.L., Pearlman J., Pieramici D.J., Rabena M.D., Castellarin A.A., Nasir M.A., Giust M.J., Wendel R., Patel A. (2006). Intravitreal bevacizumab (Avastin) in the treatment of proliferative diabetic retinopathy. Ophthalmology.

[121] Ricci F., Bandello F., Navarra P., Staurenghi G., Stumpp M., Zarbin M. (2020). Neovascular age-related macular degeneration: Therapeutic management and new-upcoming approaches. Int. J. Mol. Sci..

[122] Stewart M.W. (2012). The expanding role of vascular endothelial growth factor inhibitors in ophthalmology. Mayo Clin. Proc..

[123] Schlenker M.B., Thiruchelvam D., Redelmeier D.A. (2015). Intravitreal anti-vascular endothelial growth factor treatment and the risk of thromboembolism. Am. J. Ophthalmol..

[124] VanderVeen D.K., Cataltepe S.U. (2019). Anti-vascular endothelial growth factor intravitreal therapy for retinopathy of prematurity. Semin. Perinatol..

[125] Day S., Acquah K., Mruthyunjaya P., Grossman D.S., Lee P.P., Sloan F.A. (2011). Ocular complications after anti-vascular endothelial growth factor therapy in Medicare patients with age-related macular degeneration. Am. J. Ophthalmol..

[126] Palmer N., Jacobs B., Shetty T., Dimaras H., Hajrasouliha A.R., Jusufbegovic D., Corson T.W. (2021). Patient preferences in retinal drug delivery. Sci. Rep..

[127] Sulaiman R.S., Basavarajappa H.D., Corson T.W. (2014). Natural product inhibitors of ocular angiogenesis. Exp. Eye Res..

[128] D’Angelo G., Duplan E., Boyer N., Vigne P., Frelin C. (2003). Hypoxia up-regulates prolyl hydroxylase activity: A feedback mechanism that limits HIF-1 responses during reoxygenation. J. Biol. Chem..

[129] Campochiaro P.A. (2015). Molecular pathogenesis of retinal and choroidal vascular diseases. Prog. Retin. Eye Res..

[130] Kelly B.D., Hackett S.F., Hirota K., Oshima Y., Cai Z., Berg-Dixon S., Rowan A., Yan Z., Campochiaro P.A., Semenza G.L. (2003). Cell type-specific regulation of angiogenic growth factor gene expression and induction of angiogenesis in nonischemic tissue by a constitutively active form of hypoxia-inducible factor 1. Circ. Res..

[131] Campochiaro P.A., Aiello L.P., Rosenfeld P.J. (2016). Anti-vascular endothelial growth factor agents in the treatment of retinal disease: From bench to bedside. Ophthalmology.

[132] Sulaiman R.S., Merrigan S., Quigley J., Qi X., Lee B., Boulton M.E., Kennedy B., Seo S.Y., Corson T.W. (2016). A novel small molecule ameliorates ocular neovascularisation and synergises with anti-VEGF therapy. Sci. Rep..

[133] Basavarajappa H.D., Lee B., Lee H., Sulaiman R.S., An H., Magana C., Shadmand M., Vayl A., Rajashekhar G., Kim E.Y. (2015). Synthesis and biological evaluation of novel homoisoflavonoids for retinal neovascularization. J. Med. Chem..

[134] Sulaiman R.S., Quigley J., Qi X., O’Hare M.N., Grant M.B., Boulton M.E., Corson T.W. (2015). A simple optical coherence tomography quantification method for choroidal neovascularization. J. Ocul. Pharmacol. Ther..

[135] Chen Q., Ma J.X. (2017). Canonical Wnt signaling in diabetic retinopathy. Vis. Res..

[136] Rubsam A., Parikh S., Fort P.E. (2018). Role of inflammation in diabetic retinopathy. Int. J. Mol. Sci..

[137] Hoesel B., Schmid J.A. (2013). The complexity of NF-kappaB signaling in inflammation and cancer. Mol. Cancer.

[138] Gilmore T.D. (1999). The Rel/NF-kappaB signal transduction pathway: Introduction. Oncogene.

[139] Hoffmann A., Natoli G., Ghosh G. (2006). Transcriptional regulation via the NF-kappaB signaling module. Oncogene.

[140] Park M.S., Choi S., Lee Y.R., Joo H.K., Kang G., Kim C.S., Kim S.J., Lee S.D., Jeon B.H. (2016). Secreted APE1/Ref-1 inhibits TNF-alpha-stimulated endothelial inflammation via thiol-disulfide exchange in TNF receptor. Sci. Rep..

[141] Rawlings J.S., Rosler K.M., Harrison D.A. (2004). The JAK/STAT signaling pathway. J. Cell Sci..

[142] Batliwala S., Xavier C., Liu Y., Wu H., Pang I.H. (2017). Involvement of Nrf2 in ocular diseases. Oxid. Med. Cell Longev..

[143] Dong L., Lin T., Li W., Hong Y., Ren X., Ke Y., Zhang X., Li X. (2021). Antioxidative effects of polypyrimidine tract-binding protein-associated splicing factor against pathological retinal angiogenesis through promotion of mitochondrial function. J. Mol. Med..

[144] Cui Y., Xu X., Bi H., Zhu Q., Wu J., Xia X., Qiushi R., Ho P.C. (2006). Expression modification of uncoupling proteins and MnSOD in retinal endothelial cells and pericytes induced by high glucose: The role of reactive oxygen species in diabetic retinopathy. Exp. Eye Res..

[145] Kowluru R.A., Chan P.S. (2007). Oxidative stress and diabetic retinopathy. Exp. Diabetes Res..

[146] Deshpande A.D., Harris-Hayes M., Schootman M. (2008). Epidemiology of diabetes and diabetes-related complications. Phys. Ther..

[147] Kim Y.W., Byzova T.V. (2014). Oxidative stress in angiogenesis and vascular disease. Blood.

[148] Park I.S., Mahapatra C., Park J.S., Dashnyam K., Kim J.W., Ahn J.C., Chung P.S., Yoon D.S., Mandakhbayar N., Singh R.K. (2020). Revascularization and limb salvage following critical limb ischemia by nanoceria-induced Ref-1/APE1-dependent angiogenesis. Biomaterials.

[149] Ahmed S.M., Luo L., Namani A., Wang X.J., Tang X. (2017). Nrf2 signaling pathway: Pivotal roles in inflammation. Biochim. Biophys. Acta Mol. Basis Dis..

[150] Kobayashi E.H., Suzuki T., Funayama R., Nagashima T., Hayashi M., Sekine H., Tanaka N., Moriguchi T., Motohashi H., Nakayama K. (2016). Nrf2 suppresses macrophage inflammatory response by blocking proinflammatory cytokine transcription. Nat. Commun..

[151] Kim K.C., Kang K.A., Zhang R., Piao M.J., Kim G.Y., Kang M.Y., Lee S.J., Lee N.H., Surh Y.J., Hyun J.W. (2010). Up-regulation of Nrf2-mediated heme oxygenase-1 expression by eckol, a phlorotannin compound, through activation of Erk and PI3K/Akt. Int. J. Biochem. Cell Biol..

[152] Wei Y., Gong J., Yoshida T., Eberhart C.G., Xu Z., Kombairaju P., Sporn M.B., Handa J.T., Duh E.J. (2011). Nrf2 has a protective role against neuronal and capillary degeneration in retinal ischemia-reperfusion injury. Free Radic. Biol. Med..

[153] Xu Z., Wei Y., Gong J., Cho H., Park J.K., Sung E.R., Huang H., Wu L., Eberhart C., Handa J.T. (2014). NRF2 plays a protective role in diabetic retinopathy in mice. Diabetologia.

[154] Lenox A.R., Bhootada Y., Gorbatyuk O., Fullard R., Gorbatyuk M. (2015). Unfolded protein response is activated in aged retinas. Neurosci. Lett..

[155] Bao L., Li J., Zha D., Zhang L., Gao P., Yao T., Wu X. (2018). Chlorogenic acid prevents diabetic nephropathy by inhibiting oxidative stress and inflammation through modulation of the Nrf2/HO-1 and NF-kB pathways. Int. Immunopharmacol..

[156] Blasiak J., Piechota M., Pawlowska E., Szatkowska M., Sikora E., Kaarniranta K. (2017). Cellular senescence in age-related macular degeneration: Can autophagy and DNA damage response play a role?. Oxid. Med. Cell Longev..

[157] Silva L.L., Lambert-Cheatham N., Stratford R.E., Quinney S.K., Corson T.W., Kelley M.R. (2021). Oral APX3330 treatment reduces L-CNV lesions in a preclinical mouse model and confirms Phase 2 DR/DME clinical dose with sufficient distribution to human retina using PBPK modeling. Invest. Ophthalmol. Vis. Sci..

[158] Fishel M.L., Cheng H., Shahda S., Kelley M.R. APX3330 Drug Development for Clinical Trials Targeting APE1/Ref-1 in Pancreatic Cancer [abstract]. Proceedings of the AACR-NCI-EORTC International Conference: Molecular Targets and Cancer Therapeutics.

[159] Oshima Y., Oshima S., Nambu H., Kachi S., Hackett S.F., Melia M., Kaleko M., Connelly S., Esumi N., Zack D.J. (2004). Increased expression of VEGF in retinal pigmented epithelial cells is not sufficient to cause choroidal neovascularization. J. Cell Physiol..

[160] Xanthoudakis S., Smeyne R.J., Wallace J.D., Curran T. (1996). The redox/DNA repair protein, Ref-1, is essential for early embryonic development in mice. Proc. Natl. Acad. Sci. USA.

[161] Kelley M.R., Fishel M.L. (2021). Personal Communication.

